# TAMs in the Gynecological Tumor Microenvironment: Insights from Cross-Cancer Studies for Immunotherapy

**DOI:** 10.3390/cancers18091372

**Published:** 2026-04-25

**Authors:** Ruixi Li, Hanyue Liang, Hao Chen, Runjia Weng, Quan Ding, Ziqiu Cai, Shirui Wang, Yulin Li

**Affiliations:** 1 Main Campus, Chengdu University of Traditional Chinese Medicine, Chengdu 610032, China; 2 School of Medical and Life Sciences, Chengdu University of Traditional Chinese Medicine, Chengdu 611137, China; 3 College of Medical Technology, Chengdu University of Traditional Chinese Medicine, Chengdu 611137, China; 4 School of Acupuncture and Tuina, Chengdu University of Traditional Chinese Medicine, Chengdu 611137, China; 5 Wuxi School of Medicine, Jiangnan University, Wuxi 214122, China; 6 School of Clinical Medicine, Chengdu University of Traditional Chinese Medicine, Chengdu 611137, China

**Keywords:** tumor-associated macrophages, gynecological cancers, tumor microenvironment, immunosuppression, therapeutic resistance, targeted therapy

## Abstract

Tumor-associated macrophages (TAMs) within the tumor microenvironment (TME) play a central role in tumor progression and therapeutic resistance in gynecological malignancies, including ovarian cancer (OC), cervical cancer (CC), and endometrial cancer (EC). This review systematically summarizes common regulatory mechanisms and tumor-specific variations in TAMs across these three malignancies, emphasizing the dual-origin developmental trajectories of tissue-resident macrophages and monocyte-derived macrophages, the CCL2-CCR2 and CSF1-CSF1R core signaling axes, and the regulation of TAMs’ functional polarization by hypoxia and metabolic reprogramming. Furthermore, the molecular mechanisms through which TAMs mediate immunosuppression and therapeutic resistance via physical barrier construction, metabolic competition, and antigen presentation impairment are analyzed, and the specific characteristics of the peritoneal microenvironment in OC, HPV-driven mechanisms in CC, and hormonal regulation in EC are compared. Therapeutic strategies targeting TAM recruitment, survival, and phenotypic reprogramming are discussed, along with TAM markers, and may provide a theoretical foundation and clinical indications for overcoming immune therapeutic resistance in gynecological malignancies.

## Simple Summary

Gynecological cancers including ovarian, cervical, and endometrial cancers often resist chemotherapy and immune therapy. This review examines how tumor associated macrophages promote tumor growth and treatment failure across these three cancers. We describe shared biological features including dual cellular origins from tissue resident and bone marrow derived cells, key molecular signaling pathways, and metabolic changes involving oxygen deprivation and nutrient competition. We also identify cancer specific variations such as the abdominal cavity environment in ovarian cancer, viral infection driven changes in cervical cancer, and hormone mediated effects in endometrial cancer. The review explains how these macrophages create immunosuppressive conditions through physical barriers, metabolic interference, and impaired immune recognition. We discuss therapeutic approaches targeting macrophage recruitment, survival, and functional reprogramming. These insights may inform strategies to overcome treatment resistance in gynecological malignancies.

## 1. Introduction

Gynecologic malignancies encompass ovarian cancer (OC), cervical cancer (CC), and endometrial cancer (EC). OC is the eighth most common cancer in women worldwide, and it has the highest mortality rate among gynecological cancers. The most common histological subtype is epithelial ovarian cancer (EOC), which originates mainly from the epithelium of the fallopian tube or the ovary. Approximately 80% of cases present with advanced-stage (FIGO stages III–IV) disease characterized by intraperitoneal spread, malignant ascites, and abdominal mass [1]. The standard management approach is cytoreductive surgery followed by platinum–taxane chemotherapy, which yields initial response rates of 80%. However, platinum resistance is frequently driven by molecular diversity, BRCA1/2 mutations, and an immunosuppressive peritoneal tumor microenvironment (TME). As a result, approximately 75% of advanced-stage patients experience recurrence within 2 years. Five-year overall survival is also dismal at 10–40% [2]. The overall survival benefits of poly(ADP-ribose) polymerase (PARP) inhibitors and bevacizumab for patients with BRCA mutations and homologous recombination deficiency (HRD) are poor despite their impact on PFS. Furthermore, OC shows low sensitivity to immune checkpoint blockade, with only 8–9% of patients achieving an objective response due to the immunosuppressive TME dominated by tumor-associated macrophages (TAMs) [3].

CC is the fourth most common cancer among women worldwide. Continuous contamination by high-risk human papillomavirus (HPV) is the primary cause of squamous cell carcinoma and adenocarcinoma. Given widespread HPV vaccination and screening programs in high-income countries, the global burden remains concentrated in low- and middle-income countries [4]. Nonetheless, recent years have seen therapeutic advances, including fertility-preserving surgery and sentinel lymph node biopsy for early-stage disease. Standard care for locally advanced CC includes concurrent chemoradiotherapy in addition to PD-1 inhibitors. Median overall survival for metastatic or recurrent disease is over 24 months when treated with a combination of immunotherapy and targeted agents [5]. However, recurrence and treatment resistance are important clinical challenges. Integrated HPV shifts the host genome, creates an immunosuppressive TME, and leads to epigenetic reprogramming, driving disease relapse and treatment failure.

Globally, EC is the sixth most common cancer among women, with growing rates of obesity and aging populations contributing to its rising prevalence. Many patients requiring hysterectomy have a good prognosis, as postmenopausal bleeding typically leads to early-stage diagnosis. Patients with advanced-stage disease, on the other hand, face a poorer prognosis, with five-year survival rates of 15% [6]. The first-line standard therapy for advanced EC is currently a combination of immune checkpoint inhibitors and chemotherapy, which significantly improves the outcomes in patients with MMRd/MSI-H disease. However, approximately 30% of patients relapse within the first year. In addition, those with mismatch repair-proficient/microsatellite stable (MMRp/MSS) tumors gain limited immunity from immunotherapy. This disease is further characterized by a lack of predictive biomarkers. Overall survival advantage remains to be confirmed in prospective studies [7].

OC, CC, and EC have different etiologies and pathogeneses; however, TAMs appear to play a significant role in all three. Through the establishment of immunosuppressive niches, they promote angiogenesis and extracellular matrix (ECM) remodeling, contributing to resistance to treatment [8]. The anatomical proximity and shared pelvic TME of these three cancers offer a unique opportunity to systematically compare the functional differences between their TAMs. The underlying regulatory mechanisms of TAMs in these three cancers are similar, but due to different etiologies, they have diverged functionally. Through the integrated analyses of TAMs in gynecologic malignancies, common functional mechanisms and cancer-specific differences have been elucidated. Single-cell sequencing technologies, spatial transcriptomics, and spatial proteomics now permit dissection of TAMs’ transcriptomic and epigenetic heterogeneity within preserved tissue spatial architecture to isolate conserved functional subpopulations and identify how they interact with T cells and other neighboring cells in solid tumors [9]. These methods permit cross-cancer comparisons of TAM subpopulations to identify commonalities and divergences in TAM infiltration across molecular characteristics and spatial localization, creating a molecular basis for identifying pan-gynecologic cancer biomarkers with prognostic predictive value and devising TAM-targeted therapeutic strategies.

TAMs emerge from tissues in two ways: tissue-resident macrophages (TRMs) and monocyte-derived macrophages (MDMs), which show functional plasticity. According to traditional classification criteria, they can also be categorized into two polarization states: classically activated macrophages (M1) and alternatively activated macrophages (M2). M1 exerts anti-tumor activity, whereas M2 enhances tumor progression [10]. However, this binary classification scheme is limited, as TAM phenotypes undergo dynamic changes over the course of tumor development, transitioning from a proinflammatory M1-like phenotype in early stages to an increasingly immunosuppressive M2-like phenotype in advanced stages. As such, a more continuous classification model would capture a more robust spectrum of TAMs with distinct intermediate phenotypes and functional gradients [11]. This developmental trajectory has been directly documented in OC and CC progression models, whereas corresponding longitudinal data in EC remain less comprehensive.

TAMs form intricate regulatory relationships with the TME via metabolic exchanges, signaling through exosomes, and epigenetic reprogramming. Current pan-cancer TAM single-cell atlases have systematically confirmed the link between TAMs’ heterogeneity and ICI efficacy [12]. Universally, TAMs are biologically similar, but they exhibit unique tissue context dependencies in gynecologic malignancies. In OC, functional differences in TAMs across peritoneal fluid, parietal peritoneum, and omentum show embryonic origin dependence, driving intraperitoneal-restricted metastasis, unlike most solid tumors [13]. In CC, TAMs show remarkable evidence of M2-TAMs. HPV oncoproteins E6/E7 drive TAM transformation toward M2-like phenotypes through the PI3K/Akt-HIF-1α and STAT3 pathways, with infiltration density increasing stepwise from cervical intraepithelial neoplasia to invasive cancer [14]. TAMs in EC contribute to an immunosuppressive TME, and their functional characteristics are associated with their molecular subtypes. In CN-low/p53wt and CN-high/p53abn subtypes of EC, TAMs correlate with low tumor mutation burden and restricted immune cell infiltration, core stromal components of immune exclusion phenotypes [15]. In summary, gynecologic tumor TAMs demonstrate multi-level regulation of macrophage functional plasticity through organ TME, hormonal background, and pathogen infection, explaining shared mechanisms underlying the limited efficacy of current immunotherapy in gynecologic tumors while revealing potential mechanisms accounting for inter-cancer differences.

To move beyond parallel descriptions, this review adopts a comparative dimensional framework examining TAM biology across four axes: ontogenetic origins of TRMs versus MDMs, core signaling pathways, such as CCL2-CCR2, CSF1-CSF1R, and TGF-β, metabolic programming involving hypoxia, lactate, lipids, and etiological constraints specific to the peritoneal microenvironment in OC, HPV oncogenesis in CC, and hormonal cyclicity in EC. Within this framework, differences in anatomical origin, hormonal dependence, and immune TME among OC, CC, and EC confer TAMs with distinct polarization states and functional characteristics while constituting an ideal system for dissecting TAM plasticity. Nevertheless, these divergent etiologies—environmental–genetic interactions in OC, viral oncogenesis driven by HPV in CC, and hormonal dysregulation in EC—give TAM subpopulations distinct functional identities that challenge the feasibility of pan-gynecologic therapeutic strategies. Certain TAM subsets, such as CD163^+^Tim4^+^ TRMs driving omental metastasis in OC, appear to lack functional equivalents in the solid epithelial microenvironments of CC or the hormone-cycling endometrium of EC. Consequently, targeted depletion of ontogenically restricted subsets may offer limited translational value across cancer types, necessitating etiology-stratified rather than anatomically unified approaches.

This review explores emerging evidence of common regulatory principles and cancer-specific differences in TAMs across gynecologic malignancies through cross-cancer comparisons of TAMs’ biological characteristics in OC, CC, and EC. Based on this comparative framework, we propose etiology-stratified interventions informed by cross-cancer comparisons encompassing TAM depletion, recruitment blockade, and phenotypic reprogramming. Focusing on cancer-specific mechanisms including embryonic-origin-dependent functional differences in peritoneal compartments of OC, HPV-E6/E7-mediated stepwise immunosuppressive transformation in CC, and hormonal and ERRα metabolic axis regulation in EC offers insights that may inform strategies to address immunotherapy resistance.

## 2. TAMs in the Gynecological TME

### 2.1. Common Regulatory Framework of TAMs in the Gynecological TME

#### 2.1.1. Development of TAMs

In OC, CC, and EC, the developmental origins of TAMs follow common biological principles across two distinct lineages: TRMs and MDMs [16]. This dual-origin mechanism represents a shared basis for TAM pool construction in the gynecologic TME and influences the dynamic evolution of the tumor immune TME. TRMs colonize various tissues of the female reproductive tract, including the ovary, endometrium, and cervix, during early embryonic development. They also possess self-renewal capacity independent of adult hematopoiesis [17]. MDMs originate from hematopoietic stem cells in adult bone marrow, differentiate into monocytes in circulation, and subsequently infiltrate tumor tissues to mature [18]. TAMs from these two lineages do not exist in isolation but rather form a dynamic, interactive network during tumor progression to collectively shape TAMs’ functional heterogeneity. During early tumor stages, omental tissue-resident macrophages (CD163^+^Tim4^+^ TRMs) are the predominant macrophage population. They drive tumor metastasis and invasive progression by promoting cancer stem cell (CSC) characteristics and epithelial–mesenchymal transition (EMT) [19]. As tumor invasiveness increases, MDM recruitment increases through THBS1+ MDSC precursor differentiation, and MDMs gradually become an important component of the TAM pool [20]. The proportionally similar interrelation between TRMs and MDMs governs the dynamic equilibrium of macrophage compositions during gynecologic tumor progression.

Gynecologic tumor-associated TRMs primarily originate from yolk sac primitive hematopoiesis, expand through the fetal liver, and subsequently colonize tissues. During early embryonic development, yolk sac hematopoiesis begins at approximately E8.25 (E8.25 in mice or gestational weeks 3–4 in humans) as the hemogenic endothelium undergoes endothelial to hematopoietic transition to generate erythroid–myeloid progenitors (EMPs). These EMPs differentiate into pre-macrophages locally within the yolk sac or enter the fetal liver after E9.5 to expand, differentiate into immature TRMs, and migrate to various tissues and organs for colonization [21]. Three TRM subpopulations exist in uterine tissues (TLF+, MHC-IIhi, and CCR2+), among which the TLF+ subpopulation exhibits the lowest monocyte dependency and self-renewal maintenance [22]. In murine cervical tissues, they exist as YsM (F4/80+FOLR2++) subpopulations with robust proliferative and self-renewal capacities within tissues [23]. These embryo-derived macrophages typically display anti-inflammatory phenotypes and participate in tissue homeostasis maintenance and post-injury repair [24]. Under TME reprogramming, they transform into phenotypes that support tumor growth. CD163^+^Tim4^+^ TRMs with embryonic origins and self-renewal capacity in OC promote cancer cells’ acquisition of cancer-stem-cell-like characteristics and become key TME components driving invasive metastasis [19]. This finding provides a theoretical foundation for therapeutic strategies targeting embryo-derived macrophages.

MDMs originate from the bone marrow hematopoietic system. Compared to TRMs, MDMs demonstrate greater phenotypic plasticity. During differentiation, circulating monocytes gradually their lose proinflammatory characteristics and gain anti-inflammatory traits. They also lose surface markers CD36 and CD11b and upregulate the expression of HLA-DR, CD4, and CD68 [25]. During tumor progression, MDM to TRM ratios are reversed. TRM predominates in early tumors and is mainly distributed in marginal regions, while MDM gradually increases in advanced tumors and tends to accumulate in the center [26]. Functionally, TRMs are most often involved in tumorigenesis and early TME formation, while MDMs reorganize the tumor microenvironment, sustain immunosuppression, and aid tumor cell proliferation [27]. MDMs are more effective in immune system suppression and metabolic reprogramming. The proportion of MDMs in tumors is also associated with poor patient survival [28]. Functional markers such as TREM2, CD163, CD204, and SPP1 are highly expressed by MDMs. These markers work together to promote gynecologic tumors’ malignant progression via metabolic reprogramming, angiogenesis promotion, and stromal remodeling [29]. As such, MDMs are not just important complements of the TAM pool but are also key effector cells of malignant tumor phenotypes.

Studies using single-cell RNA sequencing technologies have identified shared principles of TAM development in gynecologic tumors. Kirschenbaum et al. used time-resolved single-cell transcriptomics (Zman-Seq) to trace the differentiation trajectories of monocytes occurring within the TME. They showed that monocyte differentiation does not occur through simple binary transitions but rather through multi-stage continuous development involving a succession of monocytes, monocyte-derived macrophages, transitional TAMs, and mature TAMs, with transcriptomic characteristics undergoing gradual dynamic remodeling in response to tumor exposure duration [30]. These findings challenge the traditional binary model and demonstrate the continuous and plastic nature of TAM development. Yin et al. studied single-cell sequencing data from 111 gynecologic malignancies and identified 11 subpopulations of TAMs, including pro-angiogenic (Angio-Mac, NFKB1+/VEGF+), interferon-activated (IFN-Mac_CXCL9, CXCL9/10/11+), immunomodulatory, and lipid-associated [31]. Classification of these subpopulations provides a molecular basis for understanding the functional heterogeneity of TAMs in the gynecologic TME as well as a rationale for targeted therapeutic strategies.

Spatial transcriptomics has also been used to elucidate the connection between TAMs and their locations. According to findings from stereo-Seq data, TAMs are spatially organized at tumor-invasive fronts. These TAMs are mainly protumor macrophages, and their spatial organization correlates with high recurrence risk in patients [32]. In gynecologic tumors, TAMs at different developmental stages may occupy particular spatial niches to form functional cellular networks. Pro-angiogenic TAM populations are enriched in perivascular regions, while populations with specialized metabolic properties accumulate in hypoxic regions [33]. This spatial zonation pattern may be prevalent in the gynecologic TME, suggesting that TAM function depends not only on their developmental origins, but also their precise regulation by spatial niche.

In gynecologic tumors, TAMs originate from TRMs and MDMs, undergoing continuous multi-stage differentiation and forming spatially zoned distributions. Critical gaps persist in current research, as the molecular switches governing proportional transitions between TRMs and MDMs remain undefined. It is also unclear whether niche-based targeting strategies can overcome existing immunotherapy resistance. These ontogenetic differences explain why CD163^+^Tim4^+^ TRMs are therapeutically targetable in OC but lack functional equivalents in CC and EC, highlighting the limitations of pan-gynecologic macrophage depletion strategies. Elucidation of these mechanisms will provide important theoretical support for developing next-generation TAM-targeted therapeutic strategies.

#### 2.1.2. Core Signaling Axes Mediating TAM Recruitment and Survival

TAM recruitment, survival, and functional maintenance in the gynecologic malignant TME are governed by multilayered signaling networks. Multiple molecular pathways are involved in TAM regulation, such as CXCL12–CXCR4 axis-mediated myeloid cell recruitment, HIF-1α-regulated hypoxic adaptive survival, and Notch signaling-mediated cell fate determination. Among these, the CCL2–CCR2, CSF1–CSF1R, and TGF-β–SMAD signaling axes and their clinical value in OC, CC, and EC have been focal points.

The CCL2–CCR2 signaling axis: The CCL2 and CCR2 signaling axis is the core pathway mediating TAM recruitment to tumor sites. In OC, activation of this axis is correlated with peritoneal tumor dissemination. The upregulation of CCL2 expression in OC cells due to β-catenin signaling activation may lead to the recruitment of peripheral blood monocytes and induce M2-like TAM polarization [34]. Recruited TAMs later secrete large quantities of CCL2, which acts on tumor cells through CCR2 and activates JAK/STAT3 signaling in these cells to promote tumor progression [34]. In CC, CCL2 and CSF-1 have a synergistic effect on the promotion of directional migration and infiltration of PBM to tumor sites [35]. Targeting the CCL2–CCR2 axis has been proposed as a way to block monocyte recruitment at tumor sites in cervical cancer (CC), which would limit the infiltration of immunosuppressive TAMs and offer an approach to immunotherapy in CC. In EC, ERRα activates PTPMT1 transcriptionally to enhance cardiolipin synthesis, augmenting mitochondrial oxidative phosphorylation and ROS production. ROS facilitates CCL2 secretion through NF-κB activation and TAM polarization. Clinical analysis demonstrates that CCL2 expression and infiltration of M2-like TAM markers are significantly higher in advanced-stage (FIGO III–IV) patients compared to early-stage patients, indicating the important role of CCL2 in linking metabolic reprogramming and an immunosuppressive TME [36].

The CSF1–CSF1R signaling axis: The CSF1 and CSF1R signaling axis regulates TAM differentiation, survival, and function. CSF-1 is primarily secreted by tumor cells and stromal cells. Upon binding to CSF1R on monocyte/macrophage surfaces, it activates downstream PI3K/AKT and extracellular signal-regulated kinase (ERK) signaling pathways, promoting monocyte differentiation into TAMs and maintaining their survival [37]. In OC, CSF1–CSF1R signaling regulates TAM infiltration density and also promotes IL-10 and TGF-β secretion by maintaining M2-like polarization status [38]. Therapeutic regimens combining anti-CSF1R monoclonal antibody emactuzumab with PD-L1 antibody atezolizumab have demonstrated responses in patients with advanced solid tumors [39]. EC patients with elevated baseline plasma CSF1 levels exhibited significantly inferior overall survival (OS) and PFS when receiving combination therapy, with hazard ratios (HRs) of 3.55 and 2.68, respectively. This finding suggests that CSF1 may regulate TAM recruitment and function [40].

The TGF-β signaling axis: During tumor progression, TGF-β remodels the immune TME through classical SMAD-dependent pathways and nonclassical pathways, promoting TAM polarization toward protumor phenotypes. In OC, aberrant activation of TGF-β signaling pathways closely correlates with TAMs’ functional polarization. TGF-β promotes TAM polarization toward M2-like phenotypes by suppressing inflammatory functions. TGF-β synergizes with IL-10 to jointly maintain TAMs’ immunosuppressive functions [41]. In the TME of CC, the TGF-β signaling axis regulates TAMs’ functional phenotypes through complex intercellular communication networks. Single-cell sequencing studies have revealed TGF-β signaling communication between cancer-associated fibroblasts (CAFs) and TAMs by binding ligands TGF-β1 and TGF-β3 to receptors TGF-βR1 and TGF-βR2 [42]. In EC, TAMs predominantly display protumor M2-like phenotypes, with their polarization process driven by cytokines including IL-4, IL-13, and IL-10. One of the main immunosuppressive factors secreted by M2-like TAMs is TGF-β. In normal endometrial tissues, TGF-β signaling largely exerts tumor-suppressive effects and maintains tissue homeostasis. Disruption of SMAD2 and SMAD3 gives rise to endometrial tissue dysfunction, infertility, and uterine cancer [43].

The key network associated with the recruitment, survival, and M2 polarization of TAMs in OC, CC, and EC appears to involve the CCL2–CCR2, CSF1–CSF1R, and TGF-β–SMAD signaling axes. To date, most studies have focused on blockade strategies targeting individual axes, with little systematic elucidation of cross-regulatory mechanisms across all three axes. Further research is needed on the regulatory mechanisms of signaling axes specific to spatiotemporal outcomes and new intervention paradigms to target dual metabolic–immune nodes. While CSF1R inhibitors show efficacy across all three types of cancer, optimal combinations differ, including anti-angiogenics for OC, anti-viral or immunotherapy for CC, and metabolic modulators for EC.

#### 2.1.3. Metabolic Regulation Driving, Inducing, and Polarizing TAMs

Metabolic abnormalities in the TME include hypoxia, lactate build-up, lipid enrichment, and amino acid metabolism disorder. While these metabolic changes represent general features of malignant tumor cell growth across solid malignancies, studies on OC, CC, and EC confirm that they also serve as important signals for regulating TAM recruitment, polarization, and functional remodeling of TAMs. As such, elucidating the molecular mechanisms through which TME metabolic abnormalities regulate TAM phenotypic switching is particularly important in developing tumor immunotherapeutic strategies targeting TAM metabolic reprogramming.

Hypoxic TME regulation and the impact on TAM recruitment and phenotypic remodeling: TME hypoxia is common in solid tumors and may regulate TAM recruitment, phenotypic polarization, and functional remodeling through multiple mechanisms. Metabolic alterations due to lack of oxygen are important for regulating phenotypic macrophage and T cell polarization.

Metabolic profiling reveals distinct bioenergetic commitments between polarization states in macrophages. Evidence from pan-cancer studies and general macrophage biology indicates that M1-like TAMs mainly rely on glycolysis for energy supply, while M2-like TAMs preferentially take up energy through oxidative phosphorylation (OXPHOS) and fatty acid oxidation (FAO) [44]. Direct validation in gynecologic malignancies supports the conservation of these metabolic phenotypes in OC, CC, and EC. Studies across multiple solid tumor types demonstrate that MDMs are an important component of TAMs, with most of their subpopulations exhibiting high hypoxic activity. Metabolic reprogramming of TAMs by tumors is mainly seen in MDM subpopulations, and Gene Set Enrichment Analysis (GSEA) has revealed that certain TAM subpopulations possess high glycolytic activity [45]. In OC patients, TAMs’ relative content is increased in patients with high hypoxia scores, and TAMs are one of the major expressing cells for hypoxia-inducible factor-1α (HIF-1α). HIF-1α expression is correlated with CD163, a characteristic marker of M2-like TAMs [46].

Additionally, nuclear factor-κB (NF-κB) activation in TAMs under hypoxic conditions enhances their immunosuppressive capacity [47]. The STAT3 signaling pathway also participates in hypoxia-mediated TAM regulation. Hypoxia induces IL-6 secretion in TAMs, activating the JAK–STAT3 signaling pathway and promoting protumor molecule expression, such as vascular endothelial growth factor (VEGF) and matrix metalloproteinases (MMPs) [48]. Focusing on neoplastic cells as the architects of microenvironmental hypoxia, recent findings illuminate active mechanisms in macrophage recruitment and functional manipulation. Hypoxia induces substantial ZEB1 expression in CC cells, which initiates a dual mechanism. First, CCL8 upregulation promotes TAM recruitment. Second, direct activation of CD47 transcription enhances tumor cell surface expression of this “don’t eat me” signal [49]. Additionally, hypoxia in EC causes tumor tissue necrosis, generating cellular debris and releasing inflammatory mediators that recruit peripheral blood macrophages into the tumor microenvironment. Hypoxia-induced factors, including IL-10 and TGF-β in endometrial cancer, promote TAM polarization toward M2-like states [50]. Beyond these regulatory mechanisms, experimental systems incorporating both malignant and myeloid populations have uncovered critical intercellular communication circuits. Hypoxic cervical cancer cells secrete exosomes enriched with ZEB1, which transfer this transcription factor to macrophages and activate STAT3 signaling to drive polarization toward M2-like/SIRPα+ phenotypes. These polarized TAMs subsequently engage tumor cells through the CD47-SIRPα axis to inhibit macrophage phagocytic function, completing the functional remodeling process [49].

Lactate-induced M2-like polarization mechanisms in TAMs: While lactate has long been recognized as the terminal metabolite of glycolysis under hypoxic conditions, recent studies have revealed its central role as a signaling molecule in regulating TAM polarization.

Lactate functions as a direct reprogramming signal for TAMs. Broadly characterized in solid malignancies, lactate can directly reprogram TAMs toward M2-like TAMs. These macrophages secrete immunosuppressive cytokines and promote Treg differentiation, contributing to the development of a TME conducive to tumor growth [51]. Direct mechanistic validation in gynecological malignancies encompasses three distinct molecular routes. Through Gpr132 mediation in ovarian cancer, lactate significantly inhibits polarization toward M1-like phenotypes, resulting in the downregulation of transcription and protein expression for M1-like markers CD68, CD80, and CD86 alongside proinflammatory factors. Lactate may also induce polarization toward M2-like phenotypes, resulting in increased expression and secretion of M2-like TAM markers CD163, CD209, CD206, and CD30 in a dose-dependent manner [52]. In CC, lactate enters macrophages via monocarboxylate transporter 1 (MCT1) and elevates the H3K18la histone lactylation levels. This epigenetic modification promotes TAM polarization toward M2-like phenotypes while inhibiting M1-like polarization through the targeted upregulation of GPD2 expression [53]. In EC, lactate generated by tumor cell aerobic glycolysis induces TAM polarization toward M2-like phenotypes. Lactate at concentrations of 5 to 10 mM can induce polarization over time, and in vivo experiments have confirmed that blocking lactate production or IL-6 signaling can inhibit M2-like polarization and tumor progression, exhibiting synergistic effects when combined with chemotherapy [54].

Beyond these malignancies, lactate metabolism regulates TAM differentiation and reconstructs communication networks between TAMs and other cells in the TME through activation of SPP1 and other signaling pathways. This mediates TAM involvement in tumor immune evasion [55]. Lactate further promotes TAM transformation toward M2-like phenotypes by suppressing mitochondrial function, forming a positive feedback loop of immunosuppression [56]. Clinical observations in CC reveal that H3K18la levels and macrophage alterations align with progression from a normal epithelium to precancerous lesions and subsequently squamous cell carcinoma. Inhibiting lactate secretion or uptake reverses this polarization effect [53].Tumor-cell-centric evidence reveals that in OC, Myc protein overexpressed in tumor cells enhances HIF1α transcriptional activity by inhibiting HIF1α degradation and promoting its nuclear translocation, driving aerobic glycolysis and the secretion of substantial lactate [57]. Beyond direct mechanistic validation, multiple critical signaling pathways and epigenetic regulatory routes exist for lactate-induced TAM polarization toward M2-like phenotypes in gynecologic tumors.

Lipid metabolism mechanisms regulating TAM M2-like polarization: Due to abnormal tumor cell proliferation, the TME features substantial lipid accumulation, proving an ample material foundation for lipid metabolic reprogramming in TAMs.

In the lipid-enriched TME, enhanced CD36-mediated lipid uptake leads to substantial lipid accumulation in TAMs, driving FAO/OXPHOS metabolic reprogramming. Intracellular oxidative stress induced by FAO mediates TAM polarization toward protumor M2-like phenotypes through activation of the JAK1–SHP–-STAT6 pathway, promoting TAM infiltration, proliferation, and protumor functions [58]. Lipid accumulation affects TAM differentiation and activation, and enhanced fatty acid uptake and metabolism drive metabolic reprogramming in OC. Circular RNA ATP2B4 can regulate M2-like macrophage polarization through the miR-532-3p/sterol regulatory element-binding transcription factor 1 axis, and sterol regulatory element-binding transcription factor 1 is a key transcription factor involved in lipid metabolism and M2-like TAM polarization [59]. Evidence from tumor-cell-secreted metabolic factors demonstrates that OC malignant ascites are enriched with substantial lipids, among which polyunsaturated fatty acids (PUFAs) constitute critical factors driving TAM polarization toward M2-like phenotypes. PUFAs inhibit Rho family small G protein A guanosine triphosphatase activity, resulting in the downregulation of nuclear Yes-associated protein 1 (YAP1), a key transcription factor in the Hippo signaling pathway. Inhibition of this signal reprograms TAMs into M2-like phenotypes [60]. Findings based on co-culture systems and cellular interaction models reveal that in the TME of CC, long-chain fatty acids released by tumor cells and CAFs can be taken up by CD36 and fatty-acid-binding proteins (FABPs) on the TAM surface. They are then transported into mitochondria via carnitine palmitoyltransferase 1 (CPT1) to participate in FAO. Through this process, characteristic markers of M2-like TAMs are upregulated, while the activation of proinflammatory signaling pathways is inhibited [61]. In EC, the core lipid metabolism gene LIPG can induce TAM transformation toward M2-like phenotypes by regulating lipid homeostasis as well as the Notch and Jak–Stat pathways while simultaneously shaping an immunosuppressive TME to form a cycle between lipid metabolism disorders and TAM protumor polarization [62].

Amino acid metabolism mechanisms regulating TAMs: In solid tumors, amino acids are metabolic substrates and signaling molecules in the TME, driving TAM recruitment, inducing their transformation toward M2-like phenotypes, and regulating their polarization through multiple mechanisms. In this way, they influence tumor progression and immunotherapy responses. Direct evidence in OC, CC, and EC validates these conserved mechanisms. Furthermore, tumor cells actively reshape amino acid availability to orchestrate TAM behavior. In OC, when glutamine synthetase (GS) expression levels decrease in tumor cells, glutaminase (GLS1) increases and glutamine transporter expression is upregulated, enhancing glutamine uptake and catabolism. Simultaneously, tumor cells secrete IL-10 and N-acetylaspartate (NAA), inducing TAM transformation into M2-like phenotypes [63]. In CC, the glutamine transporter SLC25A22 is highly expressed, promoting glutamine enrichment and shaping an immunosuppressive TME. Clinical samples indicate that high SLC25A22 expression is associated with the degree of infiltration of M2-like TAMs and tumor resistance to immunotherapy [64]. In EC, high levels of GLS1 expression lead to greater infiltration of a large number of TAMs into tumor tissues, creating an immunosuppressive TME and strengthening TAMS’ protumor effects.

cMyc-dependent glutamine metabolic alterations further promote TAM infiltration [65], and tryptophan metabolism also regulates TAMs in the gynecologic TME. In OC, under IL6 regulation, tumor cells convert tryptophan into kynurenine (KYN) through the TDO2 enzyme. KYN activates the aryl hydrocarbon receptor (AhR) to drive TAM infiltration and induce their polarization toward M2-like phenotypes while simultaneously upregulating PD-L1 expression. Indoleamine 2,3-dioxygenase 1/tryptophan 2,3-dioxygenase 2 (IDO1/TDO2) inhibition can reduce TAM infiltration and KYN levels [66]. In CC, tryptophan metabolism regulates TAMs in two ways. IFN-γ-IDO1-mediated tryptophan metabolic reprogramming induces autophagy in CC cells through KYN accumulation, enhancing TAMs’ phagocytic function and regulating their polarization toward M1-like phenotypes with high CD80/CD86 expression. This metabolic regulatory axis can effectively activate macrophage antitumor activity and inhibit CC growth [67].

In EC, tryptophan metabolic enzymes IDO1, TDO2, and interleukin 4 induced 1 (IL4I1) activate the AhR signaling pathway to regulate TAM recruitment and polarization. High TDO2 and IL4I1 expression drives TAMs to polarize and accumulate toward CD163^+^ M2-like phenotypes, whereas IDO1 indirectly affects TAM function by shaping inflammatory phenotypes, mediating TAM-related immunosuppressive effects [68]. Interactions between distinct cell populations within the TME further modulate metabolic amino acid TAM control. Mesenchymal stem/stromal cells derived from CC (CeCa-MSCs) induce macrophages to upregulate IDO expression, a key enzyme in tryptophan metabolism, through high IL-10 expression, promoting macrophage polarization toward M2-like TAMs. IDO-mediated tryptophan metabolic reprogramming synergizes with IL-10 to further reinforce the immunosuppressive functions of M2-like TAMs [69]. Beyond glutamine and tryptophan, various other amino acids participate in TAM recruitment, polarization regulation, and formation of an immunosuppressive TME through similar metabolic reprogramming mechanisms.

Evidence from cross-cancer analyses indicates that non-apoptotic cell death pathways also determine tumor immunity. While these mechanisms have been established across diverse solid tumors, expression profiling of cuproptosis-related long non-coding RNAs has been used to stratify tumors into distinct immune subtypes. According to a tumor immune dysfunction and exclusion algorithm, high-cuproptosis-activity tumors exhibit elevated major histocompatibility complex class II expression and increased CD8^+^ T cell infiltration, alongside significantly improved immunotherapy response rates [70]. Similarly, ferroptosis regulates tumor immunogenicity through glutathione depletion and lipid-peroxidation-dependent mechanisms, with long non-coding RNAs and microRNAs fine-tuning this pathway through the targeted modulation of glutathione peroxidase 4 [71]. These metabolism-driven non-apoptotic cell death pathways govern the tumor immune microenvironment across solid malignancies, with implications for OC, CC, and EC based on shared metabolic principles.

Metabolic abnormalities in the TME, including hypoxia, lactate accumulation, lipid enrichment, and amino acid metabolic disorders, drive TAM polarization toward M2-like phenotypes. However, most studies employ bulk cell-level analyses or single-timepoint sampling, and evidence from metabolomic and transcriptomic profiles at single-cell resolution to understand how spatiotemporal dynamics and TME metabolic heterogeneity determine macrophage phenotypic plasticity remains sparse. Future research should integrate single-cell multiomics technologies to construct dynamic regulatory networks encompassing TME metabolism, immunity, and epigenetics and use lineage tracing technologies to track the phenotypic evolution of macrophages during metabolic TME alterations. Likewise, verifiable intervention nodes could be used to develop combination immunotherapy regimens targeting metabolic reprogramming. A metabolic comparison revealed that lactate blockade may offer broader efficacy across gynecologic cancers than lipid-targeting strategies, which show greater context dependency.

#### 2.1.4. Functional Consequences of TAMs Driving Malignant Progression in Gynecological Tumors

Through the aforementioned molecular mechanisms, including developmental plasticity, signaling axis regulation, and metabolic reprogramming, TAMs progressively transform into protumor functional phenotypes within the TME and systematically participate in the regulation of malignant biological behaviors encompassing tumor proliferation, invasion, metastasis, and angiogenesis.

Tumor Proliferation and Stemness Maintenance: TAMs regulate cell proliferation and tumor stemness maintenance in OC, CC, and EC through multidimensional signaling networks. In OC, embryo-derived resident CD163^+^Tim4^+^ omental macrophages function as the TRM subset of TAMs and exist in close proximity to tumor cells to regulate their proliferative activity. It is through this process that the TME is established in the early malignant progression of OC [19]. MYBL2 activates CCL2 expression in OC to promote monocyte differentiation into TAMs. These polarized TAMs secrete various cytokines to activate tumor-stemness-related signaling pathways, enhancing CSCs’ self-renewal capacity and consequently driving malignant proliferation [72]. In CC, cytokines secreted by TAMs, including IL-6, IL-10, and IL-8, activate downstream proliferative signaling pathways to stimulate tumor cell proliferation and increase tumor volume [73]. Under hypoxic conditions, oncostatin M (OSM), secreted by CC cells, induces macrophage polarization toward the M2-like phenotype through the mammalian target of rapamycin complex 2 (mTORC2) signaling pathway. These polarized macrophages subsequently secrete IL-6, VEGF, and other factors to further promote tumor cell proliferation [74]. In EC, miR-21 secreted by tumor cells induces monocyte polarization toward M2-like TAMs. These M2-like TAMs exhibit a high expression of arginase 1 (Arg-1) and CD206 but low expression of inducible nitric oxide synthase (iNOS) and CD86. They also promote tumor cell proliferation through the secretion of IL-6 and other cytokines [75].

Invasion, Metastasis, and EMT Conversion: TAMs drive invasion and metastasis in OC, CC, and EC by regulating ECM remodeling, EMT-related signaling pathways, and the immunosuppressive TME. MAs in OC are enriched with TAMs and TGF-β1. This TME promotes cancer cell migration, and TGF-β1 secreted by TAMs induces EMT to enhance tumor cells’ invasive capacity [76]. CCL18 enhances OC cell migration and invasion by activating the PI3K/Akt/GSK3β/Snail signaling pathway, promoting peritoneal dissemination and distant metastasis [77]. In CC, matrix metalloproteinase 9 (MMP-9), secreted by TAMs, promotes tumor cell invasion and metastasis. Current evidence supports the pro-metastatic role of the MMP family in CC progression [78]. Regarding lymphangiogenesis, TAMs interact with tumor cells to induce lymphatic vessel formation in CC, further expanding routes for tumor metastasis [79]. Significant infiltration of CD163^+^CD204^+^ M2-like TAMs is strongly correlated with STAT3/NF-κB signaling pathway activation. These two factors constitute a central axis that regulates malignant progression in CC. This regulatory axis drives tumor EMT conversion by suppressing the epithelial marker E-cadherin and activating the mesenchymal marker Vimentin and the EMT transcription factor SNAIL. Simultaneously, it upregulates molecules such as MMP9 and VEGFα to degrade the ECM and promote angiogenesis, directly enhancing the invasive and metastatic capacity of CC [80]. In EC tissues, lactate produced by tumor cells through aerobic glycolysis induces macrophage polarization toward the M2-like phenotype. IL-6 secreted by these macrophages further promotes EMT conversion and neovascularization in tumor cells, enhancing their invasive and metastatic capabilities [54].

Angiogenesis and Metabolic Symbiosis: TAMs provide essential nutritional support and TME conditions for the growth and progression of gynecological tumors by secreting pro-angiogenic factors, regulating tumor metabolic reprogramming, and establishing metabolic symbiotic loops. TAMs secrete pro-angiogenic factors including VEGF and platelet-derived growth factor (PDGF) to directly stimulate vascular endothelial cell proliferation and migration, promoting tumor neovascularization. Exosomes derived from TAMs promote endothelial cell proliferation, increase tumor vascular density, and enhance blood supply [81]. M2-like macrophage-derived exosomes downregulate hypoxia inducible factor-1α Inhibitor expression, activating HIF-1α and vascular endothelial growth factor A signaling to promote angiogenesis. This mechanism effectively induces choke vessel remodeling and neovascularization, enhancing skin flap survival. Notably, these pro-angiogenic effects are substantially attenuated by 2-methoxyestradiol, a specific HIF-1α inhibitor [82]. In OC, M2-like TAMs can directly contact endothelial cells to enhance vascular barrier function and reduce vascular permeability, limiting MA formation. They also maintain tumor vascular network stability by secreting VEGF and other factors to support sustained tumor growth [83]. In CC, TAMs activate the NF-κB signaling pathway by secreting inflammatory factors such as TNF-α and IL-1β, indirectly upregulating pro-angiogenic factor expression [84]. In EC, CXCL3^+^ macrophages are specific to tumor tissues and exhibit characteristics of M2-like TAMs with the highest angiogenesis scores. These cells participate in cytokine-stimulated response pathways, inflammatory reactions, and other processes to promote tumor angiogenesis. This subset represents a late stage of macrophage differentiation, with related genes progressively upregulated during pseudotemporal progression to regulate key pathways, including MAPK cascades and neutrophil degranulation [85].

**Figure 1:**
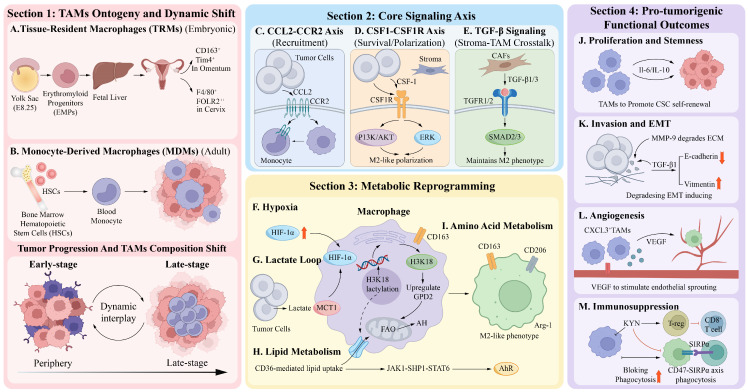
**Ontogeny, regulatory signaling, and metabolic reprogramming of tumor-associated macrophages (TAMs) in the gynecologic cancer microenvironment.** The multifaceted role of TAMs in ovarian, cervical, and endometrial cancer is illustrated through four integrated dimensions: Section 1: **Ontogeny and Dynamic Shift: (A)** Embryo-derived tissue-resident macrophages (TRMs) origin, such as CD163^+^Tim4^+^ omental macrophages or F4/80+FOLR2++ cervical macrophages; (**B**) Bone marrow-derived macrophages (MDMs) recruited from circulating monocytes; Section 2 **Core Signaling Axis: (C)** The CCL2-CCR2 axis mediating monocyte recruitment; (**D**) The CSF1-CSF1R axis activating PI3K/AKT and ERK pathways to sustain M2-like polarization; (**E**) TGF-β-SMAD2/3 signaling facilitating crosstalk between CAFs and TAMs; Section 3 **Metabolic Reprogramming: (F)** Hypoxia-induced HIF-1α activation promoting immunosuppression; (**G**) The lactate loop where tumor-derived lactate drives H3K18 lactylation and M2 polarization via MCT1; (**H**) CD36-mediated lipid metabolism and fatty acid uptake fueling the JAK1-SHP1-STAT6-AhR axis; (**I**) Amino acid metabolism, including the KYN-AhR axis driving TAM recruitment; Section 4: **Pro-tumorigenic Functional Outcomes: (J)** TAM-secreted IL-6/IL-10 enhancing tumor stemness and proliferation; (**K**) MMP-9 and TGF-β1 secretion driving invasion and EMT; (**L**) CXCL3+ TAMs stimulating angiogenesis through VEGF production; (**M**) Immunosuppression mediated by the KYN pathway and the CD47-SIRPα signaling axis.

TAMs acquire functional heterogeneity through developmental plasticity in the gynecological TME. Via signaling axes and metabolic reprogramming mechanisms, these cells ultimately transform into core functional phenotypes that promote tumor proliferation, invasion, metastasis, and angiogenesis (Figure 1). These malignant biological behaviors, together with a TAM-mediated immunosuppressive TME and therapeutic resistance, contribute to the complex regulatory networks underlying gynecological tumor progression. They also provide actionable intervention targets for developing combination therapeutic strategies targeting TAMs.

### 2.2. Cancer-Specific TAM Regulatory Networks

OC, CC, and EC differ in lesion location and etiological factors, leading to distinct local TMEs. Consequently, TAMs polarize differently across these three gynecological malignancies. MA flow, persistent HPV infection, and cyclical changes in sex hormones influence the functional orientation of macrophages. Investigating these mechanisms provides essential support for the design of subsequent TAM-targeted therapeutic strategies.

#### 2.2.1. OC

The anatomical location of the ovary adjacent to the peritoneal cavity constitutes the structural basis for the route of peritoneal dissemination and metastasis in OC. The peritoneal cavity provides a specific TME for tumor progression, with the hallmark formation of MAs. Within MAs, TAMs can be classified into two major subpopulations: tumor-enriched macrophages (TeMs) and ascite-enriched macrophages (AeMs). AeMs exhibit significant functional deficiencies with reduced expression levels of HLA-II molecules, but they significantly express S100A family pro-tumor proteins and chemokines such as CCL2. AeMs form an interaction network with desmin (DES)-positive mesothelial cells in MAs through signaling pathways such as CXCL12–CXCR4, collectively shaping and maintaining the pro-tumorigenic inflammatory microenvironment of Mas [86]. Unlike most solid tumors, TAMs in OC are not only infiltrated into primary lesions but are also more widely distributed in metastatic sites, such as MAs and the omentum. Single-cell transcriptome sequencing analysis reveals that myeloid cells are significantly more enriched in liquid tissues than in solid tissues, with macrophages constituting the predominant myeloid component across all examined tissues. This has been validated in the ID8 mouse model [87].

The omentum is the primary target organ for peritoneal metastasis in EOC. It is also the most common site of recurrence. Omental colonization-state TAMs are predominantly CD163^+^Tim4^+^ TRMs. Notably, this subpopulation is ontogenically restricted to the peritoneal microenvironment of OC and appears to lack functional equivalents in the solid epithelial microenvironments of CC or the hormone-cycling endometrium of EC. These cells induce tumor cells to acquire CSC-like characteristics and promote distant dissemination by activating the JAK–STAT signaling pathway. In contrast, parietal peritoneal adhesion-state TAMs are mainly composed of the CD163^+^Tim4^−^ subset derived from monocytes, supporting primary tumor growth [19]. The spatial distribution and functional differentiation of these two TAM subpopulations influence the immunological TME heterogeneity of peritoneal metastasis in OC. Peritoneal mesothelial cells form a monolayer structure covering the abdominal organs. The underlying matrix consists of fibronectin, type I collagen, type IV collagen, and laminin. The cell surface is covered by a pericellular matrix rich in hyaluronic acid (HA). This structure endows mesothelial cells with dual functions. Surface HA can form a natural barrier that hinders OC cell adhesion. However, secreted ECM molecules can mediate tumor cell anchoring through β1 integrin or CD44 to construct tumor niches [88]. MAs can also stimulate peritoneal mesothelial cells to secrete angiopoietin-like 4 (ANGPTL4). This molecule facilitates the migration and recruitment of blood monocytes, which are TAM precursor cells, from the periphery [89].

#### 2.2.2. CC

Given the central role of HPV infection in CC initiation and progression, this pathogen regulates TAMs during lesion evolution in the TEM, with characteristic stepwise transformation. As cervical lesions progress from low-grade squamous intraepithelial lesions (LSIL) to high-grade squamous intraepithelial lesions (HSILs) and invasive cervical squamous cell carcinomas (CESCs), TAM infiltration patterns and functional phenotypes of undergo substantial alterations. Single-cell sequencing shows that at the LSIL stage, M1-like/M2-like ratios are high. Overall, M1-like/M2-like ratios steadily decline from the HSIL to the CESC stage, and M2-like TAM infiltration significantly increases. Clinical cohort studies have shown a gradual increase in HPV E6/E7 mRNA copy numbers during disease progression. Moreover, CD163^+^ M2-like TAMs in HSIL tissues are significantly enriched [90]. Large-scale meta-analyses support this evolutionary model, as high stromal densities of CD163^+^ M2-like TAM are closely associated with advanced FIGO (International Federation of Gynecology and Obstetrics) stage and lymph node metastasis [79]. HPV-positive CC tissues show notable enrichment of M2-like TAMs, which have heightened expression of antigen presentation-related genes such as CD74 and human leukocyte antigen A [91]. HPV infection increases macrophage quantity and, more importantly, alters their functional phenotypes. HPV-positive cells secrete increased levels of GCSF, IL-6, and IL-8, which synergistically promote tumor-associated neutrophil and M2-like TAM recruitment [92]. In HPV16 and HPV18-positive cervical intraepithelial neoplasia (CIN) tissues, PSGL-1 expression levels are closely related to infection status, lesion grade, immune infiltration level, and clinical prognosis, further elucidating the early actions of viral factors in TAM functional reprogramming [93]. Culture supernatants obtained from CC cells directly induce THP-1 macrophage polarization into M2-like TAMs, suggesting that tumor cells can reprogram TAM functionality through the secretion of soluble factors [94]. The regulatory mechanisms acting upstream on these soluble factors and cell–cell interactions are increasingly being unveiled as direct targets of HPV oncoproteins.

#### 2.2.3. EC

In EC, macrophage regulation by estrogen and progesterone is complex and hormone-specific. According to classical models, ER and PR classical signaling pathways have regulatory functions. However, emerging studies show that ERRα and heme metabolism also mediate TAM polarization and functional reprogramming. Estrogen is responsible for the transition of TAMs from the M1-like to the M2-like phenotype in EC through the regulation of Zinc Finger Protein 626 (ZNF626) and Ste20-like Kinase (SLK) and the downregulation of RING Finger and WD repeat domain 3 (RFWD3) [95]. Immune TME remodeling by hormones is an important mechanism of EC progression, and targeting these downstream genes may be a novel approach for precision therapeutics. ERRα, a key metabolic regulator, transcriptionally activates PTPMT1 expression by directly binding to the promoter region of protein tyrosine phosphatase mitochondrial 1 (PTPMT1), promoting cardiolipin synthesis and enhancing OXPHOS activity. ROS accumulation activates NF-κB pathways and causes CCL2 secretion to induce M2-like TAM recruitment [36]. TAM regulation through progesterone is associated with heme metabolism. In patients with progesterone resistance, elevated expression of key enzymes in heme synthesis drives TAM polarization toward M2-like phenotypes and promotes immunosuppressive factor secretion [96]. Clinical–pathological correlations further support these mechanisms. CD163^+^ TAM infiltration correlates with progesterone resistance in patients with endometrial atypical hyperplasia or endometrioid carcinoma, and infiltration density is inversely correlated with PR expression levels [97].

Based on the TCGA molecular classification framework, TAM infiltration patterns and functional polarization exhibit the following characteristics. In POLE-mutant EC, TAMs demonstrate no distinct polarization bias toward either M1-like or M2-like phenotypes. CNH (TP53-mutant) subtypes predominantly harbor M2-like TAMs with significantly elevated infiltration of PD-L1^+^CD68^+^ TAMs. NSMP subtypes exhibit the lowest TAM infiltration levels without distinct polarization characteristics. CNL subtypes show unclear polarization and functional biases [98]. CNH EC can be further classified into immune-hot and immune-cold subtypes. The former demonstrates TAM co-enrichment with pro-tumorigenic immune cells, whereas the latter exhibits immune-desert phenotypes with minimal TAM infiltration. Granzyme M (GZMM) has prognostic value for CNH subtypes but not for CNL subtypes, suggesting that TAM functional regulatory mechanisms differ across molecular subtypes [99].

Existing studies have delineated distinct TAM regulatory landscapes across OC, CC, and EC. Peritoneal metastatic lesions in OC demonstrate spatial functional segregation between TeMs and AeMs. HPV oncoproteins in CC drive stepwise transformation of TAMs from M1-like to M2-like phenotypes. EC is regulated by the hormone–metabolism axis. However, from a cross-cancer comparative perspective, whether these three TME pressures, including peritoneal fluid flow, persistent viral infection, and cyclic hormonal fluctuations, determine macrophages’ functional fate through shared metabolic reprogramming mechanisms remains unclear. Furthermore, limited clinical responses to TAM-targeted interventions may be due to the neglect of network dependencies formed between TAMs and stromal cells, such as mesothelial cells, rather than purely cell-autonomous defects, warranting further investigation.

## 3. The TAM-Mediated Immunosuppressive TME and Drug Resistance Mechanisms in Gynecological Tumors

### 3.1. TAM-Mediated Construction of the Immunosuppressive TME

#### 3.1.1. Physical Barriers

Mechanisms characterized across diverse solid tumors demonstrate that TAMs systematically abrogate antitumor immune responses by constructing physical barriers to reshape the spatial architecture of the TME. In OC, CC, and EC, this process involves ECM remodeling, aberrant angiogenesis, and the formation of specific spatial structures. In OC, CD163^+^ M2-like TAMs secrete transforming growth factor beta induced protein (TGFBI), which mediates ECM remodeling and aberrant fibrosis within the OC TME, establishing physical barriers that impede immune cell infiltration [100]. In response to TGF-β, CAFs accumulate ECM components that create barriers to T cell infiltration and action. Moreover, tumor necrosis factor alpha induced protein 6 (TNFAIP6) induced by TGF-β promotes TAM polarization to M2-like phenotypes, while TGF-β-activated CAFs produce IL-6 and IL-11, which enhance the immunosuppressive TME [101]. Through physical isolation, TAMs surpass ECM barriers by regulating the spatial distribution of immunosuppressed cells. To promote the local accumulation of Tregs and neutrophils via chemokine secretion, sialic acid-binding immunoglobulin-like lectin 9-positive tumor-associated macrophages (Siglec-9^+^TAMs) form physical clusters of immunosuppressive cell populations. This cellular subpopulation also has adhesive connections with CD8^+^ T cells and transmits immunosuppressive signals through contact [102].

In the hypoxic cancer microenvironment, TAMs form defined spatial structures. TAMs release IL-10 to activate specificity protein 1 (Sp1) in lymphatic endothelial cells (LECs), which induces CCL1 expression. This creates a positive feedback loop that encourages more TAMs and tumor cells to join, eventually resulting in an LVEM containing a network of lymphatic vessels wrapped in TAMs. This structure creates physical barriers due to spatial occupancy effects [103]. TAMs’ impaired phagocytic ability also contributes to a physical–functional barrier for immune evasion. Stanniocalcin 1 (STC1) is a macrophage phagocytic checkpoint molecule with an inverse expression correlation with the long non-coding RNA lymph node metastasis associated suppressor (LNMAS) in CC tissues. Downregulation of LNMAS expression leads to transcriptional activation and overexpression of STC1 in CC cells, which directly suppresses the phagocytic function of TAMs. This establishes a functional physical barrier that mediates immune suppression and enables immune evasion by CC cells [104]. In EC, TAMs secrete pro-angiogenic factors to participate in tumor angiogenesis regulation, and aberrant tumor vasculature is a physical barrier that obstructs immune cell infiltration. Cytokines secreted by TAMs can induce immune checkpoint molecule expression, and angiogenic pathways co-express with immune checkpoint molecules across different EC subtype subpopulations, synergistically reinforcing the immunosuppressive effects of physical barriers [105]. Huang et al. used a three-dimensional physical barrier model of a disintegrable supramolecular gelatin hydrogel (DSG) formed through host–guest interactions to demonstrate that M2-like TAMs within the barrier secrete high levels of VEGF and accelerate oxygen consumption. This synergizes with the spatial characteristics of the barrier that limit oxygen diffusion to create a hypoxic environment, further promoting M2-like TAM polarization and accumulation. Through positive feedback loops, this process drives and reinforces the formation of an immunosuppressive TME and malignant progression in EC [106].

#### 3.1.2. Metabolic Competition

TAMs engage in nutritional competition through metabolic competition, depriving the TME of essential substrates such as glucose and amino acids while simultaneously accumulating immunosuppressive metabolites including lactate and KYN. TAMs employ a mechanism in the TME associated with immune exhaustion and metabolic deprivation, potentially aiding in the construction of an immuno-suppressive TME. TAMs also deplete tryptophan and yield KYN and other metabolites in amino acid metabolism, creating an immunosuppressive TME. In OSCC, KYN metabolic pathways associated with M1-like TAMs hinder T cell proliferation via metabolic competition, creating a negative metabolic feedback loop for tumor immune evasion [107]. In CC, TAMs collaborate with IDO-1-mediated tryptophan metabolic pathways to participate in immune suppression. IDO-1 is highly expressed in CC tissues and induces immune suppression by depleting local tryptophan and producing immunosuppressive metabolic products, which are correlated with poor patient prognosis. Targeted inhibition of IDO-1 can reduce its expression and activity, restoring local antitumor immune responses in CC [108].

In EC, M2-like TAMs secrete kynureninase (KYNU) and its metabolic product 3-hydroxyanthranillic acid (3-HAA), which activate endoplasmic reticulum oxidoreductin 1α (ERO1α) and the unfolded protein response pathway through superoxide dismutase 2 (SOD2)-dependent mitochondrial reactive oxygen species regulatory mechanisms, promoting tumor cell survival and stemness maintenance. Simultaneously, transcription factor 4 (ATF4)-mediated positive feedback loops are activated to amplify KYNU secretion in hypoxic TME, contributing to an immunosuppressive TME [109]. Beyond amino acid depletion, TAMs further deprive immune cells of membrane structural substrates through lipid metabolic reprogramming. They also activate pro-tumorigenic signals to reinforce immune suppression. In OC, tumor cells induce membrane cholesterol efflux from TAMs, activating the STAT6 and PI3K–mTORC2–Akt signaling pathways, enhancing IL-4 signaling, and inhibiting IFN-γ-induced gene expression. These lipid metabolic reprogramming events produce an immunosuppressive TME [110].

In CC, consensus clustering analysis based on lipid-metabolism-related genes (LMRGs) reveals that macrophage infiltration and CD68 expression (a marker of M1-like TAMs) are significantly higher in the C2 subgroup than in the C1 subgroup, and this subgroup demonstrates better prognosis. Meanwhile, CCL20 expression is significantly higher in the C1 subgroup than in the C2 subgroup, and CCL20 can induce the accumulation of immature dendritic cells (DCs) to suppress antitumor immune responses [111]. In EC, the lipid TME formed by lipid metabolic reprogramming in tumor tissues not only provides substrates for lipid metabolism in TAMs but also activates pro-tumorigenic signaling pathways mediated by monounsaturated fatty acids to further promote TAM polarization toward M2-like phenotypes. TAMs shape the immunosuppressive TME in EC through lipid metabolic regulation [112]. In glucose metabolism, TAMs achieve the dual suppression of nutritional deprivation and accumulation of immunosuppressive metabolites through competitive glucose uptake and massive lactate production. In OC, TAMs enhance glucose uptake capacity through ubiquitin D (UBD)-mediated glycolytic metabolic reprogramming, generating large quantities of metabolites such as lactate. As a core immunosuppressive metabolite, lactate synergizes with immunosuppressive factors secreted by M2-like TAMs to reduce the infiltration and activation efficiency of CD8^+^ T cells, driving the formation of an immunosuppressive TME [113]. In CC, TAMs highly express hexokinase 3 (HK3), which promotes nuclear translocation of transcription factor EB (TFEB) by binding to the mechanistic target of rapamycin (mTOR), inhibiting CD8^+^ T cell function. Furthermore, IFN-γ secreted by activated CD8^+^ T cells can induce HK3 expression in TAMs, forming a metabolically mediated negative feedback loop of immune suppression [114]. Following TAM lactate-induced polarization and senescence in EC, TAMs reinforce the immunosuppressive state of the TME by secreting immunosuppressive cytokines while simultaneously promoting malignant tumor cell progression. This establishes a lactate-based metabolic interaction cycle to produce an immunosuppressive TME [115].

#### 3.1.3. Antigen Presentation Impairment

TAMs play dual roles in regulating antitumor immune responses. However, in gynecological malignancies, TAM functional reprogramming is significantly skewed toward immunosuppressive M2-like phenotypes, and antigen presentation impairment limits ICI efficacy. In the OC TME, tumor cells highly express CD47 and interact with signal regulatory protein alpha (SIRPα), inhibiting TAMs’ phagocytic function. Clinical samples demonstrate elevated CD47 expression in OC tissues compared to adjacent normal tissues. CD47 gene knockout or treatment with anti-CD47 monoclonal antibody (mAb) can increase macrophage phagocytic indices by over 30% and effectively trigger phagocytic clearance of CD133^+^ tumor initiating cells (TICs), restoring antigen presentation function [116]. Beyond direct phagocytic inhibition, physicochemical characteristics of the TME also influence TAMs’ functional differentiation. ECM can induce monocyte differentiation into ECM-educated macrophages (MAMs), among which high-disease-associated MAMs (HD MAMs) exhibit low expression of antigen-presentation-related molecules such as human leukocyte antigen-DQA1 (HLA-DQA1) and insufficient acquisition of tumor antigens due to defective phagocytic function [117]. Meanwhile, TAM subpopulations defined by specific surface markers also inhibit antigen presentation. TAMs expressing folate receptor β (FRβ) are an immunosuppressive M2-like subpopulation characterized by high PD-L1 expression and low expression of the costimulatory molecule CD86, which can directly inhibit antigen-specific T cell proliferation and IFN-γ secretion, exacerbating antigen presentation impairment [118].

In the CC TME, secreted phosphoprotein 1-positive TAMs (SPP1^+^ TAMs) represent a specific immunosuppressive subtype that can directly impair antigen presentation capacity through the high expression of PD-L1 and IL-10. They can further exacerbate antigen presentation defects through TNFRSF18-mediated synergistic effects that induce myeloid cell maturation disorders [119]. TAMs also contribute to an immunosuppressive TME through functional abnormalities in core antigen presentation molecular processes, such as peptide-major histocompatibility complex class II (peptide-MHC II) receptor activity. In addition, TAMs can engage in specific ligand–receptor interactions with the dominant Epithelial_1 subpopulation in tumor tissues, and their spatial colocalization allows for positive feedback regulation through paracrine signals, inhibiting TAMs’ antigen presentation function [120]. Clinical intervention studies have also confirmed the central role of TAMs in immunotherapy resistance. During neoadjuvant chemotherapy combined with PD-1 blockade for CC treatment, CD74 expression in TAMs is significantly elevated, mediating antigen presentation dysfunction by inhibiting phagocytic function and promoting M2-like polarization to form an immunosuppressive TME [121].

In EC, TAMs predominantly comprise CXCL8-high, IL1B-high macrophages (CXCL8hiIL1Bhi macrophages). Compared to C3-high, IL1B-low macrophages (C3hiIL1Blo macrophages), they possess antigen presentation function in normal endometrial tissues, while TME reprogramming significantly downregulates HLA family molecule expression in TAMs, resulting in antigen presentation dysfunction [122]. Analysis of TME characteristics in immunotherapy non-responders further corroborates the clinical significance of antigen presentation impairment. The TME of immunotherapy non-responding EC patients is enriched with M2-like TAMs, and antigen processing and presentation pathways in this subtype lack activation, with no high expression of immune-activation-related molecules, directly causing antigen presentation dysfunction. Moreover, M2-like TAMs preferentially interact with non-immune cells such as endothelial cells and epithelial cells to promote angiogenesis and tumor proliferation, completely lacking antigen-presentation-related signal transmission with immune cells. This further exacerbates local immunosuppressive states [123]. TAM density in EC is increased approximately 9.4-fold compared to normal endometrial tissues, and highly expressed CD47 molecules on tumor cell surfaces interact with signal regulatory protein-α (SIRPα) on TAM surfaces to effectively inhibit phagocytic function and obstruct TAMs’ antigen presentation capacity [124].

#### 3.1.4. Cellular Network

The immunosuppressive nature of the TME is driven by intricate intercellular communication networks. Within these networks, TAMs show strong crosstalk with many other cell types. As key myeloid cells, TAMs interact with a variety of cell types, such as T cells, CAFs, endothelial cells, DCs, and neutrophils, to establish immunological barriers in tumors. Tissue-specific cellular interactions in gynecological malignancies have different effects on patient prognosis and treatment responses.

TAMs–T cells: TAMs and T cells collaboratively produce an immunosuppressive TME through sustained bidirectional communication, and their spatial proximity and functional interactions determine the inactivation state of local immune responses. In OC, TAMs form triplet niches with CD8^+^ T cells through PD-L1 expression, blocking costimulatory signal transmission between T cells and DCs and promoting the formation of an immunosuppressive TME. The terminal exhaustion state of CD8^+^ T cells conversely determines their interaction patterns with myeloid cells, and enhanced interactions between CD8^+^ T cells and TAMs in mixed inflammatory and excluded tumor types drive the TME’s transformation toward an immune-resistant phenotype [125]. CCL23 secreted by TAMs can directly induce CD8^+^ T cell exhaustion. Elevated CCL23 levels are observed in MAs and the plasma of HSGC patients, and CCL23-high tumor tissues demonstrate increased proportions of CTLA-4^+^ CD8^+^ T cells [126]. Single-cell sequencing reveals that TAMs send intercellular communication signals to Tregs with the highest frequency. CD163 expression in tumor tissues demonstrates a significant positive correlation with expression of the master transcription factor of Tregs, forkhead box P3 (FOXP3). Additionally, T-cell-derived IFN-γ may drive the transformation of TAMs toward M2-like phenotypes, suggesting that effector T cells may regulate TAM polarization [127].

In CC, TAMs and CD8^+^ T cells form functional interaction and feedback loops. TAMs promote CD8^+^ T cell exhaustion through inhibitory signals mediated by leukocyte immunoglobulin-like receptor B4 (LILRB4), and the functional decline of CD8^+^ T cells further exacerbate the degree of immunosuppression in the TME [128]. CCL22 induces TAM polarization toward M2-like phenotypes through autocrine pathways in CC. In vitro TAM models constructed using human peripheral blood MDMs co-cultured with the CC cell lines HeLa and Siha demonstrate that CCL22 knockdown reduces the mean fluorescence intensity of CD206 on TAM surfaces, whereas CCL22 overexpression upregulates CD206 expression. This confirms that CCL22 drives TAM polarization toward M2-like phenotypes in an autocrine manner. Previous studies have shown that CCL22 can recruit Tregs to tumor tissues, inhibiting antitumor immune responses [129]. Uterine CD4^+^ and CD8+ T cells display a tissue-resident memory T cell phenotype with robust expression of CD69 and CD103. These cells also exhibit selective functional dysfunction characterized by PD-1 expression but minimal levels of CTLA-4 and other inhibitory receptors. Despite sustained PD-1 expression, these tissue-resident memory T cells retain effector functions capable of producing IFN-γ, IL-17, and granzyme B. Notably, IL-17 generation is predominantly regulated by phosphorylated signal transducer and activator of transcription 3. This hybrid phenotype, which combines dysfunctional features with preserved effector capacity, enables uterine T cells to maintain immune tolerance toward the semi-allogeneic fetus while simultaneously protecting against pathogenic challenges [130].

In EC, aryl hydrocarbon receptor nuclear translocator-like (ARNTL) knockout reduces the proportion of M2-like TAMs and enhances CD8^+^ T cell activity, during which M1-like TAM markers are upregulated while M2-like TAM markers are downregulated, indicating that TAMs are closely associated with T cell functional states [131]. TAMs exhibit polarization differences in EC. M2-like TAM infiltration increases in the non-specific molecular subtype (Other) with poor prognosis, whereas the MSI-H subtype with a better prognosis is enriched with M1-like TAMs. The MSI-H subtype is also simultaneously enriched with CD8^+^ T cells and Tregs. Single-cell sequencing further reveals the presence of PD-1^+^ T cells and CD68^+^ myeloid cell populations in MSI-H tumors, indicating that TAMs and T cells participate in TME regulation through polarization imbalance and functional exhaustion [132]. In addition to contact-dependent inhibition and paracrine cytokine signaling, TAMs may further modulate T cell function through the intercellular transfer of vesicular cargo. Co-culture of CD4^+^ Jurkat T cells with exosome-mimetic nanovesicles derived from MDA-MB-231 breast cancer cells markedly elevated the expression of the proinflammatory cytokines IFN-γ and IL-17a and their transcription factors T-bet and RORC2 and reduced the expression of the anti-inflammatory cytokines IL-10 and TGF-β1 and the regulatory T cell marker FoxP3. Tumor-cell-derived vesicles can induce a proinflammatory phenotypic switch in T cells [133], indirectly supporting the hypothesis that macrophage-derived vesicles within the gynecological TME may regulate T cell immune responses through similar intracellular delivery mechanisms.

TAMs–CAFs: TAMs and CAFs are core populations of stromal cells. They form functional interaction networks that drive the establishment of the immunosuppressive TME. In OC, spatial colocalization of TAMs and CAFs is the foundation for the immunosuppressive TME. Periostin (POSTN) activates ERK/NF-κB signaling to induce tumor cells to secrete cytokines such as MCP-1 and TNF-α, promoting TAM polarization toward M2-like phenotypes. Simultaneously, POSTN activates CAFs by inducing TGF-β2 expression. TCGA data analysis demonstrates that high POSTN expression positively correlates with abundant α-SMA and FAP^+^ CAFs, and conditioned media from POSTN-pretreated CAFs exhibit enhanced pro-tumorigenic activity. These findings suggest that POSTN can simultaneously regulate both TAMs and CAFs to produce an immunosuppressive TME [134]. High-grade serous carcinoma (HGSC) is the most common and most malignant subtype of EOC. Spatial transcriptomics reveals that mesenchymal regions in HGSC are enriched with TAMs, which, together with desmoplastic CAFs, establish an immunosuppressive TME. CAFs sequester naive T cells via the CXCL12–CXCR4 axis, preventing their infiltration into malignant areas. Concurrently, TAMs suppress CD8+ T cell function through CD80/CD86-CTLA4 ligand–receptor interactions, cooperating with CAFs to facilitate immune evasion [135].

Similar malignant cooperation has been observed in CC. Tumor-cell-conditioned medium induces mesenchymal stem cells (MSCs) to differentiate into CAFs with high fibroblast activation protein (FAP) expression. These CAFs secrete IL-6 and IL-10 to drive M2-like polarization of TAMs, and FAP expression progressively increases during tumor progression [136]. These findings indicate that CAFs cooperate with TAMs through signal transduction and the STAT3–CCL2 axis to promote the formation of an immunosuppressive TME. TAMs exhibit high CD74 expression and enrichment of immunomodulatory pathways, including IL6–JAK–STAT3 and TNFα/NF-κB. CellChat analysis demonstrates that CAFs serve as central mediators through midkine (MK) signaling pathways. Furthermore, the interaction between CAFs and TAMs is significantly enhanced in tumor tissues compared to normal tissues, and both populations contribute to the establishment of an immunosuppressive TME [137]. Interactions between TAMs and CAFs in EC also demonstrate distinct immunosuppressive characteristics. The CD146+ CAF subset secretes substantial IL-10, which mediates angiogenesis and vasculogenic mimicry (VM) formation through the IL-10/JAK1/STAT3 pathway, establishing a TME with abundant blood supply [138]. This abundant vascular supply provides nutrients for tumor growth, and the accompanying local hypoxia and metabolic disturbances resulting from aberrant vascular structures can also activate hypoxia-related proinflammatory signaling pathways. These processes drive the formation of an immune-tolerant TME, whereas CD146+ CAFs cooperate with M2-like TAMs to construct an immunosuppressive TME.

**Figure 2:**
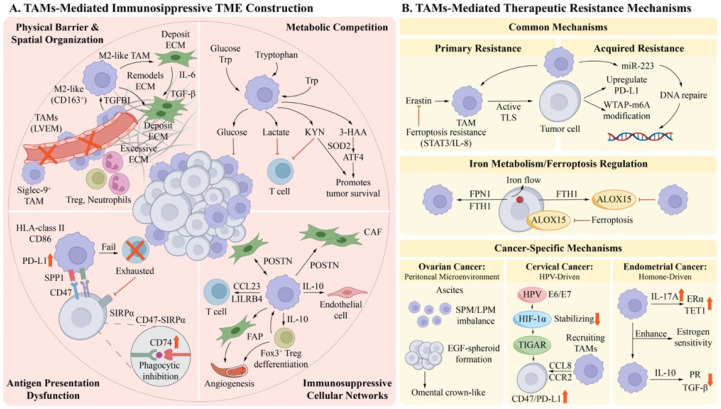
**Tumor-associated macrophage (TAM) mechanisms that produce an immunosuppressive tumor microenvironment (TME) and drive therapeutic resistance in gynecological cancers.** (**A**) **TAM-mediated immunosuppressive TME production. (1) Physical Barriers and Spatial Organization**: TAMs facilitate extracellular matrix (ECM) remodeling via TGFBI and TGF-β secretion, forming a physical barrier that restricts T cell infiltration. Siglec-9^+^ TAMs and lymphatic vessels encapsulated by TAMs (LVEM) further reinforce spatial isolation. **(2) Metabolic Competition**: TAMs deplete essential nutrients (glucose and tryptophan) and accumulate immunosuppressive metabolites such as lactate, kynurenine (KYN), and 3-hydroxyanthranilic acid (3-HAA) to inhibit T cell proliferation and promote tumor survival. **(3) Antigen Presentation Dysfunction**: The CD47-SIRPα checkpoint and elevated CD74 expression inhibit macrophage phagocytosis, while downregulation of HLA-DR and CD86 impairs antigen processing and presentation. **(4) Immunosuppressive Cellular Networks**: TAMs engage in complex crosstalk with T cells, cancer-associated fibroblasts (CAFs), and endothelial cells via the CCL23, LILRB4, and IL-10/STAT3 pathways to maintain an immune-resistant landscape. (**B**) **TAM-mediated therapeutic resistance mechanisms: (1) Common Mechanisms**: TAMs confer resistance via the pre-activation of translesion DNA synthesis (TLS), exosomal delivery of miR-223, and WTAP-mediated m6A RNA methylation. Regulation of ferroptosis through the Nrf2/FPN1 axis and IL-6/KIAA1429 pathway also contributes to acquired resistance. **(2) Iron Metabolism and Ferroptosis Regulation:** TAMs remodel iron homeostasis by activating the Nrf2 pathway in tumor cells, leading to upregulation of the iron exporter FPN1 and ferritin, which reduces intracellular labile iron levels. Additionally, M2-like TAMs secrete IL-8 via the STAT3 pathway to enhance resistance to ferroptosis-inducing agents like Erastin, while macrophage-derived exosomes deliver miRNAs that inhibit ALOX15 expression, reducing lipid reactive oxygen species (ROS) production and preventing ferroptosis-mediated cell death. **(3) Cancer-Specific Mechanisms**: In ovarian cancer (OC), the peritoneal microenvironment promotes resistance through omental crown-like structures and EGF-mediated spheroid formation. In cervical cancer (CC), HPV E6/E7 oncogenes drive TAM polarization and metabolic adaptation. In endometrial cancer (EC), TAMs enhance estrogen sensitivity via TET1-mediated epigenetic modulation and induce progestin resistance by downregulating progesterone receptors (PRs).

Through multiple mechanisms, including physical barrier construction, metabolic resource competition, antigen presentation dysfunction, and multi-cellular network interactions, TAMs systematically establish an immunosuppressive TME in OC, CC, and EC. Current research has provided a relatively comprehensive map of molecular interactions between TAMs and components such as ECM, CAFs, and T cells. Future investigations should integrate spatial transcriptomics and metabolomics technologies to construct spatiotemporal evolution maps of TAMs’ functional states and explore combined intervention strategies targeting the TAM–CAF–T cell tripartite interaction network to inform novel precise therapeutic targets to reverse the immunosuppressive TME (Figure 2).

### 3.2. TAM-Mediated Mechanisms in Therapeutic Resistance

#### 3.2.1. General Mechanisms of TAM-Mediated Therapeutic Resistance

Primary resistance: Research on solid malignancies indicates that in addition to regulating tumor cell fate through direct intercellular contact, TAMs can confer primary resistance abilities on tumor cells prior to the initiation of chemotherapy and immunotherapy through cytokine and exosome secretion as well as the induction of metabolic reprogramming. In OC specifically, TAMs activate nuclear translocation of the NF-κB p65 subunit and transcriptionally upregulate the translesion DNA synthesis key enzyme Pol η, along with its regulatory factors RAD18 and REV1, while concurrently suppressing the high-fidelity nucleotide excision repair (NER) pathway, potentially contributing to cisplatin resistance in tumor cells. Relatedly, CSC-like characteristics are enhanced given increased proportions of CD44+CD117+ cells and elevated spheroid formation capacity. Furthermore, TAMs can pre-activate the translesion DNA synthesis (TLS) pathway at the baseline to establish a drug-resistant phenotype in tumor cells in advance [139]. This TLS pathway provides a molecular foundation for the development of acquired resistance during treatment. Tumor cells under hypoxic conditions can also recruit macrophages and induce their polarization into TAMs. Exosomes secreted by TAMs deliver microRNA-223 to tumor cells, regulating phosphatase and tensin homolog deleted on chromosome ten (PTEN) and the PI3K/AKT pathway to directly confer a primary drug-resistant phenotype on tumor cells [140].

TAMs also function as critical mediators in primary resistance to CC treatment. Chemokine-like factor-associated MARVEL transmembrane domain-containing protein 6 (CMTM6) is delivered to TAMs via exosomes, activating the mTOR pathway and driving their polarization toward the immunosuppressive M2a phenotype, attenuating antitumor immunity and inducing drug resistance. M2a-polarized TAMs secrete CCL2 to produce a positive feedback loop that reinforces the immunosuppressive TME and malignant tumor progression [141]. Therefore, the CMTM6–M2a–CCL2 axis is a critical molecular network governing resistance regulation in CC. Beyond chemotherapy, TAMs also mediate primary resistance to immunotherapy. Studies have revealed that TAM density within the tumor stroma of ICI responders is significantly lower than in non-responders, and low TAM density is an independent predictor of ICI response. In the MMRd/MSI-H subtype, spatial proximity between TAMs and B cells is significantly increased, potentially compromising tertiary lymphoid structures’ antitumor function by suppressing B cell proliferation and downregulating germinal center reactions [142]. Concurrently, genetic mutations can promote primary resistance by remodeling tumor and immune interactions. The CHD4 R975H mutation activates CSC characteristics and TAM M2-like polarization, producing a vicious cycle that promotes therapeutic resistance. This mutation activates TGF-β and TNF-α signaling through NF-κB and other pathways via transcriptome reprogramming, which may upregulate the expression of the stemness markers KLF4, NANOG, and OCT4 and TAM transformation toward M2-like TAMs. The interaction between CSCs and M2-like TAMs not only produces an immunosuppressive TME, but also enhances tumor cell resistance to conventional therapy by maintaining their self-renewal capacity [143].

Acquired resistance: TAM polarization toward the M2-like anti-inflammatory/pro-tumoral phenotype represents a central mechanism mediating acquired resistance in gynecologic malignancies. Acquired resistance is a critical bottleneck limiting the efficacy of chemotherapy and radiotherapy in gynecologic malignancies. In OC, circular RNA circITGB6 is significantly overexpressed in platinum-resistant patients. It forms an RNA–protein ternary complex with insulin-like growth factor 2 mRNA-binding protein 2 and fibroblast growth factor 9 mRNA to enhance FGF9 stability, inducing TAM polarization toward the M2-like phenotype. Antisense oligonucleotides targeting circITGB6 can reverse M2-like polarization and restore chemosensitivity [144]. In patient-derived OC organoids and humanized patient-derived xenograft (huPDX) models, M2-like TAMs are selectively recruited to the TME and associated with enhanced OC cell survival through direct intercellular contact and paracrine effects, which may contribute to paclitaxel resistance [145]. These findings elucidate the critical role of TAM polarization in OC resistance, and this pattern also exists in other gynecologic malignancies.

In CC, studies demonstrate that conditioned medium from irradiated tumor cells overexpressing SEPT9 can induce the polarization of THP-1-derived macrophages toward the M2-like phenotype, characterized by elevated CD206 and Arg1 expression and reduced iNOS expression. This indicates that SEPT9 influences radiosensitivity in CC by mediating TAM polarization through miR-375 [146]. Chemotherapy itself can also promote TAM polarization through indirect mechanisms. Following cisplatin or carboplatin treatment of CC cells, the DNA damage response activates the NF-κB pathway, further increasing PGE2 and IL-6 production and enhancing the capacity to induce M2-like polarization in monocytes. These chemotherapy-induced M2-like TAMs exhibit reduced IL-12 secretion and increased IL-10 secretion, accompanied by STAT3 activation and STAT1/STAT6 inhibition, which establishes an immunosuppressive TME and leads to therapeutic resistance [147]. M2-like TAMs further promote tumor resistance through diverse signaling interaction mechanisms. In OC, co-culture of TAMs with tumor cells upregulates PD-L1 in both populations, accompanied by the activation of resistance-related genes including signal transducer and activator of transcription 3 and multidrug resistance protein 1. Silencing PD-L1 in tumor cells alone partially restores carboplatin sensitivity, but simultaneous silencing PD-L1 in both cell types yields maximal chemosensitivity, suggesting a synergistic role for TAM-derived PD-L1 in resistance [148]. Furthermore, TAMs secrete CXCL16, which binds to CXCR6 on the tumor cell surface, activating WTAP-mediated N6-methyladenosine (m6A) RNA methylation modification. This upregulates m6A methyltransferase WTAP and reader protein YTHDF1 while inhibiting demethylase ALKBH5, enhancing the expression of DNA repair proteins PARP1, BRCA1, and BRCA2 and suppressing apoptosis, which promotes tumor cell resistance to cisplatin [149]. In EC, M2-like TAMs transfer circular RNA hsa_circ_0001610 to EC cells via secretion of exosomes (EXOs). This molecule functions as a competing endogenous RNA (ceRNA) to sponge microRNA-139-5p (miR-139-5p), mitigating the inhibitory effect of miR-139-5p on cyclin B1 (CCNB1). Upregulation of CCNB1 expression accelerates G2/M phase cell cycle transition, attenuates radiation-induced DNA damage effects, and reduces radiosensitivity in EC cells [150].

TAMs precisely regulate iron metabolic homeostasis in tumor cells and intervene in ferroptosis processes. This molecular mechanism underlies acquired resistance in gynecologic malignancies. In OC, low concentrations of the ferroptosis inducer Erastin activate the STAT3 pathway to promote TAM polarization toward the M2-like phenotype and increase IL-8 secretion, enhancing the invasive and metastatic capabilities of Erastin-resistant OC cells (SKOV3 and HO8910) [151]. Bioinformatics screening identifies cytochrome b-245 beta chain (CYBB) as a key gene linking ferroptosis and macrophage infiltration. CYBB is highly expressed in OC and correlates with poor prognosis. Its knockdown significantly reduces tumor cell sensitivity to Erastin and promotes the expression of ferritin heavy chain 1 (FTH1) and ferroptosis suppressor protein 1 (FSP1) in TAMs while simultaneously facilitating TAM polarization toward the M2-like phenotype [152]. This suggests that CYBB influences the TME by regulating ferroptosis sensitivity in TAMs, indicating that CYBB holds potential value as a therapeutic target in ferroptosis induction therapy for OC.

In CC, TAMs promote therapeutic resistance by suppressing ferroptosis through exosome-mediated microRNA transfer. Targeted inhibition of arachidonate 15-lipoxygenase (ALOX15) expression reduces lipid reactive oxygen species generation and suppresses ferroptosis. Clinical data demonstrate that low ALOX15 expression correlates with TAM infiltration and poor patient prognosis [153]. TAMs can activate the nuclear factor erythroid 2-related factor 2 (Nrf2) pathway in tumor cells through paracrine signaling, upregulating iron exporter ferroportin 1 (FPN1) and ferritin expression. Iron efflux is also enhanced, increasing iron storage capacity. Furthermore, the labile iron pool level in cells is reduced, and ferroptosis is suppressed. Ultimately, this mediates therapy resistance in cancer cells.

Our multi-omics analysis suggests that Nrf2 expression is significantly reduced in CC and that low Nrf2 expression is negatively correlated with increased infiltration of CD163-positive M2-like TAMs. Single-cell sequencing coupled with immunofluorescence validation showed a decrease in Nrf2 expression with tumor stage progression and a corresponding increase in CD163 expression. Direct binding of Nrf2 to the CD163 promoter region was confirmed by chromatin immunoprecipitation sequencing [154]. These findings show that ferroptosis-related molecules and TAMs mutually regulate one another, which could be useful for TME iron-metabolism-targeted immunotherapy. TAMs in EC produce high levels of IL-6. Research has shown that IL-6 activates the JAK1/STAT3 pathway to increase KIAA1429, which mediates m6A modification of DNA damage inducible transcript 3 (DDIT3) mRNA and its degradation, reducing DDIT3 expression and leading to ferroptosis resistance in EC cells [155].

Prior to treatment initiation, TAMs confer intrinsic resistance to tumor cells by pre-activating the TLS pathway and exosome-delivered molecules. Following polarization toward the M2-like phenotype, TAMs mediate acquired resistance during treatment by regulating PD-L1 expression, m6A modification, iron metabolic reprogramming, and ferroptosis inhibition, establishing an immunosuppressive TME that persists throughout therapeutic resistance in gynecologic malignancies.

#### 3.2.2. Cancer-Specific Differences in TAM-Mediated Resistance Mechanisms

Resistance mechanisms mediated by the peritoneal microenvironment: The peritoneal microenvironment is a regional ecosystem composed of the peritoneal mesothelial cell layer, subperitoneal ECM, omental adipose tissue with its resident immune cells, and ascites. In the OC peritoneal microenvironment, TAMs are predominantly composed of immunosuppressive small peritoneal macrophages (SPMs) and immunocompetent large peritoneal macrophages (LPMs), which differ in their development, phenotype, and function. In the ascite microenvironment, the proportion of SPMs is significantly elevated, while that of LPMs is decreased. SPMs actively recruit Tregs using the high expression of chemokines (CCL17 and CCL22) and, at the same time, high expression of PD-L1, PD-L2, IDO2, TGF-β, and IL-10 to dampen the antitumor immune response to the chemotherapeutic drug cisplatin [156]. The imbalance of macrophage subsets in the ascite microenvironment is only one kind of peritoneal resistance mechanism. The omental tissue is the main target site for OC cells’ intraperitoneal metastasis and underlies TAM resistance. The milky spot structures present within the omentum are important sites for preferential colonization and growth of tumor cells. Omental fat, which is highly enhanced with the elevation of cytokines in obesity, could enhance tumor metastasis. The omentum’s adipocytes may supply tumor cells with fatty acids to provide energy. The interaction between milky spots and adipocytes sets up a unique peritoneal microenvironment that provides the structural and material basis for TAMs’ pro-metastatic and pro-resistance functions [157].

In addition to their structural roles, CD163^+^Tim4^+^ TRMs in omental tissue influence resistance regulation through specific signaling pathways. This cellular subset does not rely on MDMs and is an important component of the OC pre-metastatic niche. By activating the JAK–STAT signaling pathway, it can enhance EMT and the acquisition of CSC features in tumor cells, driving the development of therapeutic resistance [158]. Metabolic factors in MAs and the omental microenvironment, particularly lipid components, exert important regulatory effects on TAMs’ functional status. PUFAs enriched in MAs and omental conditioned medium (OCM) drive TAM polarization toward the M2-like phenotype and enhance OXPHOS metabolism through the PUFAs–YAP1 axis, promoting the formation of an immunosuppressive TME and tumor cell resistance [60]. Beyond metabolic regulation, the CSF1R signaling pathway also has critical immunosuppressive functions in the ascite microenvironment. Accumulation of CSF1R-positive TAMs is positively correlated with vascular permeability, and these cells induce vascular dysfunction and MA formation through VEGF-independent mechanisms [159]. Studies on the PUFAs–YAP1 axis and the CSF1R signaling pathway reveal the molecular mechanisms through which the peritoneal microenvironment shapes TAMs’ pro-tumoral phenotype, while metabolic reprogramming characteristics of the ascite microenvironment further amplify the complexity of this resistance pattern.

In HGSC, peritoneal effusions from platinum-resistant patients exhibit distinct hyperlipidemic characteristics. This hyperlipidemic state induces immune cell exhaustion and promotes TAM polarization toward the M2-like phenotype. Tumor cells gain the ability to enhance macropinocytosis through the high expression of Src homology 3 domain containing YSC84-like protein 1 (SH3YL1). This enables a substantial uptake of lipids from MAs (macrophages) and intensification of lipid catabolism (metabolism) to provide energy and synthetic materials for tumor cells [160]. The TME and tumor cell metabolic reprogramming help reduce sensitivity to platinum-based agents. Thus, a specific resistance pattern is established in the ascites. Crown-like patterns are found in omental metastatic lesions in advanced HGSC. These structures are apoptotic adipocytes in a protective environment formed by various types of macrophages. Patients whose omentum displays CD68-positive or CD163-positive crown-like structures have a significantly greater incidence of platinum chemotherapy resistance. Unlike TAMs in primary lesions, omental crown-like-structure-associated macrophages can induce chemotherapy resistance in tumor cells through lipid metabolic reprogramming and immunosuppressive effects [161]. The omental crown-like structures mediate drug resistance by organizing spatially, whereas TAMs in the ascite microenvironment impede drug penetration through the aggregation of tumor cells into spheroids. TAMs promote tumor spheroid formation through epidermal growth factor (EGF), which upregulates integrins and intercellular adhesion molecule 1 (ICAM-1), limiting chemotherapy drug penetration. Upregulation of a macrophage receptor with collagenous structure (MARCO) in ascite spheroids occurs through transcriptomic mensagens. MARCO is associated with immunosuppression and has demonstrated therapeutic targeting potential in models including breast cancer. Furthermore, upregulation of the OXPHOS pathway and members of heat shock protein family A (HSPA) in ascite spheroids mediate chemotherapy resistance [162].

HPV-driven therapeutic resistance mechanisms: In CC, HPV-driven TAM polarization only establishes an immunosuppressive TME but also directly mediates therapeutic resistance through specific molecular pathways. The HPV E6 oncoprotein promotes metabolic reprogramming in tumor cells through the upregulation of TIGAR expression while simultaneously inducing the infiltration of immunosuppressive cells, including Tregs and TAMs, to establish an immunosuppressive TME. TIGAR reduces intracellular reactive oxygen species levels, decreases DNA damage, and promotes DNA repair, which is associated with resistance to platinum-based agents and various other chemotherapeutic drugs [163]. HPV E6/E7 can promote metabolic reprogramming by interfering with the prolyl hydroxylase domain 2 (PHD2)–von Hippel–Lindau (VHL)–Cullin 2 (CUL2)–Elongin C (ELOC)–HIF-1α axis, resulting in elevated HIF-1α expression in CC. E6 and E7 can also induce aberrant miRNA expression, and these miRNAs can target and suppress the expression of components of the E3 ubiquitin ligase complex, including VHL and PHD2, further promoting HIF-1α-mediated metabolic adaptation [164]. Under hypoxic conditions, HIF-1α activates ZEB1 transcription through direct binding to hypoxia response elements in the proximal promoter region of ZEB1, subsequently driving ZEB1 binding to the CCL8 promoter to enhance CCL8 expression. CCL8 recruits TAMs to infiltrate hypoxic regions through the CCR2–NF-κB signaling pathway [165].

Beyond these mechanisms, leukemia inhibitory factor (LIF) in HPV-associated CC can suppress interferon-I production by plasmacytoid dendritic cells (pDCs) and block CXCL9 expression in TAMs, inhibiting tumor infiltration by CD8-positive T cells [166]. High LIF expression is associated with poor patient prognosis and tumor resistance. HPV infection can also promote the high expression of CD47 and PDL-1 on the tumor cell surface through the upregulation of lysine-specific demethylase 1 (LSD1) expression. These molecules can inhibit phagocytosis and immune clearance by TAMs, reducing the efficacy of immunotherapy and mediating therapeutic resistance [167]. In CC patients, aberrant expression of HLA class I molecules and HLA-G is associated with IL-10 expression, and dysregulated immune modulation significantly impairs antigen presentation function [168]. This mechanism participates in the formation of an immunosuppressive TME and mediates tumor resistance. Furthermore, E6 and E7 proteins can regulate the miRNA expression profile in tumor cells and exosomes, and these miRNAs can act on TAMs to reduce their antitumor activity and contribute to therapeutic resistance [169].

Hormone-driven therapeutic resistance mechanisms: The regulation of TAM polarization status by steroid hormones directly determines tumor treatment sensitivity. In EC, this mechanism involves dual layers of epigenetic modification and receptor expression regulation, serving as a representative model for in-depth analysis. In EC, TAMs predominantly exhibit an M2-like polarization state. These cells promote tumor growth, invasion, and metastasis by secreting immunosuppressive factors and facilitating angiogenesis. CD68^+^CD163^+^ TAMs activate TET1-mediated DNA hydroxymethylation by secreting IL-17A, upregulating ERα expression to enhance cancer cell sensitivity to estrogen. In EC, TAMs primarily regulate ERα rather than ERβ. This mechanism is most significant in endometrial atypical hyperplasia (EAH) and G1-stage cancer tissues, and it diminishes as tumors progress to G2–G3 stages. Furthermore, serum estradiol levels are not elevated in EC patients [170]. These findings indicate that TAMs drive tumor progression by enhancing local tissue estrogen sensitivity rather than elevating systemic hormone levels.

Furthermore, insulin upregulates ten-eleven translocation 1 (TET1) expression by activating the PI3K/AKT signaling pathway. TET1 subsequently epigenetically regulates G protein-coupled estrogen receptor (GPER) expression through DNA hydroxymethylation modifications, enhancing estrogen sensitivity [171]. TAM-induced ERα expression and TET1-mediated GPER upregulation constitute a unique dual estrogen sensitivity mechanism in EC. ERα is highly expressed in EC. It triggers mitochondrial metabolic reprogramming by transcriptionally upregulating protein tyrosine phosphatase mitochondrial 1, promoting reactive oxygen species accumulation, and activating nuclear factor kappa B signaling. It also recruits M2-like TAMs through CCL2, forming an immunosuppressive TME and mediating immunotherapy resistance. This metabolic–immune crosstalk regulatory pattern is specific to EC [36]. Progestin therapy resistance is closely associated with TAMs, and this mechanism exhibits significant cancer type specificity. Unlike common chemotherapy resistance patterns observed in other solid tumors, M2-like TAMs in EC directly downregulate PR expression in tumor cells by secreting cytokines, leading to progestin treatment failure [97,172,173]. In fertility-sparing treatment, progestin resistance is closely associated with CD163^+^ TAM infiltration. These macrophages secrete IL-10 and TGF-β to downregulate PR expression and block progestin signal transduction. Concurrently, they further attenuate the antitumor effects of progestin by altering chromatin accessibility and ECM signaling pathways. This mechanism distinguishes EC from other hormone-related tumors [97].

In OC, the peritoneal microenvironment establishes a peritoneum-specific resistance pattern through multiple mechanisms, including SPM/LPM subset imbalance, spatial organization of omental milky spots and crown-like structures, the PUFAs–YAP1 metabolic axis, and CSF1R signaling pathways. CC exhibits HPV-driven resistance. E6/E7 oncoproteins drive TAM polarization by regulating TIGAR, HIF-1α, and LIF molecules while mediating resistance to platinum-based chemotherapy and immunotherapy. EC demonstrates a unique hormone-dependent resistance mechanism. TAMs enhance estrogen sensitivity through TET1-mediated epigenetic modifications and directly downregulate PR expression to cause progestin treatment failure. These cancer-type-specific TAM resistance mechanisms provide important targets for developing precision combination therapeutic strategies.

## 4. TAM-Targeted Therapy and Precision Medicine

### 4.1. Applications and Challenges of TAM-Targeted Therapy

#### 4.1.1. Blocking TAM Recruitment and Survival

**Figure 3:**
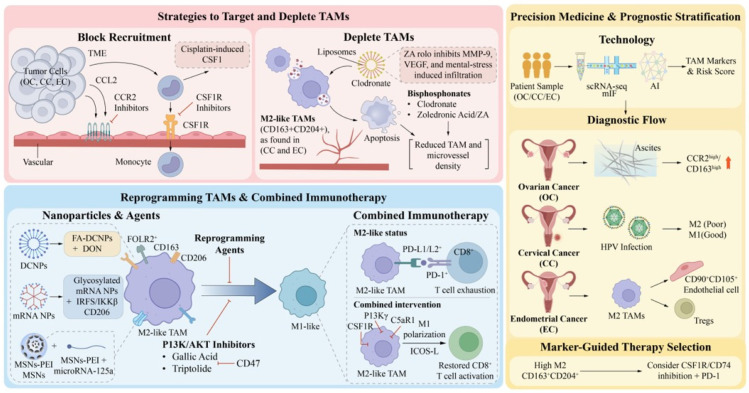
**Therapeutic strategies targeting tumor-associated macrophages (TAMs) and precision medicine applications in gynecological malignancies. Strategies to target and deplete TAMs**: These include blocking recruitment via CCL2–CCR2 or CSF1–CSF1R signaling inhibitors and direct depletion using bisphosphonates (e.g., clodronate liposomes or zoledronic acid) to reduce TAM density and angiogenesis. **TAM reprogramming and combined immunotherapy**: Using nanoparticles (e.g., FA-DCNPs, mRNA NPs) and PI3K/AKT inhibitors to polarize pro-tumor M2-like TAMs into anti-tumor M1-like phenotypes. Synergistic effects are achieved by combining TAM-targeting agents (e.g., C5aR1, PI3Ky, or CSF1R inhibitors) with PD-1/PD-L1 blockade to restore CD8^+^ T cell activity and overcome immune resistance. **Precision medicine and diagnostic flow**: Integration of single-cell RNA sequencing (scRNA-Seq), multiplex immunofluorescence (mIF), and AI-driven analysis for patient stratification. Specific prognostic markers and TME features for ovarian cancer (OC), cervical cancer (CC), and endometrial cancer (EC) are highlighted to guide personalized therapy selection.

TAMs play a pivotal role in the initiation, progression, and therapeutic resistance of various gynecologic malignancies, including OC, CC, and EC. Given TAMs’ remarkable phenotypic plasticity and pro-tumoral functions, targeted modulation of their recruitment, survival, and polarization has emerged as a critical research direction in immunotherapy for gynecologic tumors (Figure 3) (Table 1).

**Table 1:** Clinical trials of TAM-directed therapeutics in gynecologic malignancies: ovarian, cervical and endometrial cancers.

Type	Targeting	Drug	Trial	Phase	Scheme	Population	Outcomes	Ref.
OC	CSF1R	Emactuzumab	NCT01494688	I	15 mg/kg or 1200 mg IV q3w; or in combination with paclitaxel	Advanced solid tumors (including OC)	Specifically reduce immunosuppressive TAMs, but none have produced clinically relevant anti-tumor activity	[174]
			NCT02760797	I	1000 mg or 2000 mg IV q2w	Advanced solid tumors (including OC)	CD14dimCD16+ monocytes are reduced, and the activation of Ki67+CD8+ T cells is increased, but the objective clinical remission is limited	[175]
		LY3022855	NCT02718911	I	10, 15, 22.5, or 30 mg/kg IV q2w	Advanced solid tumors (including OC)	Limited clinical efficacy	[176]
	CCL2	Carlumab	NCT00537368	I	0.3, 1.0, 3.0, 10, or 15 mg/kg IV q2w	Advanced solid tumors (including OC)	Has anti-tumor activity	[177]
			NCT01204996	I	15 mg/kg IV q2w	Advanced solid tumors (including OC)	Limited tumor response	[178]
	PD-1/Ang2	Pembrolizumab + Trebananib	NCT03239145	I	Pembro 200 mg IV q3w + Trebananib 15 mg/kg IV qw	Recurrent Platinum-Resistant OC	——	[179]
	VEGF/ Ang2	Vanucizumab	NCT01688206	I	Bi-weekly (q2w) schedule: 3–30 mg/kg Weekly (qw) schedule: 10–30 mg/kg	Advanced solid tumors (including OC)	Reduced tumor angiogenesis leads to a good anti-tumor effect	[180]
	PD-1/VEGF-A	Ivonescimab (AK112/SMT112)	NCT04047290	I	10 mg/kg IV q2w	Advanced solid tumors (including OC)	Preliminary anti-tumor activity; patients who have previously received bevacizumab treatment may still benefit from it	[181]
	PD-1	Pembrolizumab + Carboplatin + Paclitaxel	NCT03126812	I/II	200 mg IV q3w/400 mg IV q6w 20 mg/kg IV q2w	Advanced solid tumors (including OC)	Achieved a 27% major pathological response rate. The median PFS and OS of major responders has not been reached, which is significantly better than that of minor responders	[182]
	PD-L1	Atezolizumab + Bevacizumab + Non-platinum chemotherapy	NCT03353831	III	Atezolizumab 840 mg IV q2w + Bevacizumab 10 mg/kg IV q2w + CT (weekly paclitaxel 80 mg/m^2^ or PLD 40 mg/m^2^) q4w	Platinum-resistant or platinum-ineligible recurrent OC (first or second relapse ≤ 6 months, or third relapse; prior bevacizumab allowed)	Failed to significantly improve recurrent OS or PFS, and the efficacy is independent of PD-L1 expression status	[183]
		Atezolizumab + Bevacizumab + Platinum-based chemotherapy	NCT02891824	III	1200 mg IV q3w 15 mg/kg IV q3w (Combination medication) 15 mg/kg IV q3w (Maintenance treatment)	Platinum-sensitive recurrent epithelial OC, one to two previous chemotherapy lines, platinum-free interval > 6 months	Failed to significantly improve PFS in ITT or PD-L1 positive populations	[184]
	IL-12	IMNN-001	NCT03393884	I/II	IMNN-001 100 mg/m^2^ IP qw × 17 (neoadjuvant 8 + adjuvant 9) + IV carboplatin/paclitaxel	Newly diagnosed FIGO stage III/IV high-grade epithelial OC	IMNN-001 combination chemotherapy showed a clinically meaningful 13-month overall survival benefit, with no systemic IL-12-related toxicity observed	[185]
	CD47/CD40	SL-172154	NCT04406623	I	Schedule 1: Days 1, 8, 15 in Cycle 1; q2w (Days 1, 15) from Cycle 2; Dose levels: 0.1, 0.3, 1.0, 3.0, 10.0 mg/kg Schedule 2: qw (Days 1, 8, 15, 22); Dose levels: 0.1, 0.3, 1.0, 3.0, 10.0 mg/kg	Advanced solid tumors (including OC)	SL-172154 is well-tolerated in platinum-resistant patients. The 3.0 mg/kg dose can achieve saturation of both CD47/CD40 targets, M1-like macrophage polarization, and CD8+ T cell infiltration. No bell-shaped dose response or severe hepatotoxicity, which are typical of traditional CD40 agonists, has been observed	[186]
CC	mTORC1/2	Sapanisertib (CB-228/TAK-228) + Metformin	NCT03017833	I	Sapanisertib 30 mg QW + Metformin 500–1500 mg/day	Advanced solid tumors (including OC)	Preliminary anti-tumor activity	[187]
	PD-L1+VEGF	Atezolizumab + Bevacizumab + Chemotherapy	NCT03556839	III	Atezolizumab 1200 mg IV q3w; Bevacizumab 15 mg/kg IV q3w; Cisplatin 50 mg/m^2^ or Carboplatin AUC 5 + Paclitaxel 175 mg/m^2^ IV q3w	M/P/R CC; Previously untreated with systemic therapy for metastatic/recurrent disease	Significantly improve PFS and OS	[188]
	VEGF	Bevacizumab + Chemotherapy	NCT00803062	III	15 mg/kg IV q3w	R/P/M CC; GOG PS 0–1; adequate organ function	Studies have confirmed the efficacy and tolerability of anti-angiogenic therapy in advanced CC	[189]
	PD-L1	Atezolizumab + Bevacizumab	NCT02921269	II	Bevacizumab: 15 mg/kg IV q3w Atezolizumab: 1200 mg IV q3w	Advanced CC 1–2 previous lines of therapy in the advanced setting Including at least one containing bevacizumab	It did not reach the preset mitigation endpoint, and its safety was consistent with the known characteristics of the drug, but two cases of serious neurological adverse events were observed	[190]
		Pembrolizumab + platinum-based chemotherapy (paclitaxel + cisplatin/carboplatin) ± bevacizumab	NCT03635567	III	Pembrolizumab: 200 mg IV Q3W for up to 35 cycles. Chemotherapy: Paclitaxel 175 mg/m^2^ + cisplatin 50 mg/m^2^ or carboplatin AUC 5, IV Q3W for 6 cycles. Bevacizumab (optional): 15 mg/kg IV Q3W	Persistent, recurrent, or metastatic CC, adenocarcinoma, adenosquamous carcinoma, or squamous cell carcinoma not amenable to curative treatment. ECOG performance status 0 or 1	Can significantly prolong the patient’s OS	[191]
		Nivolumab	NCT02257528	II	Nivolumab was administered intravenously at a dose of 3 mg/kg every 2 weeks (q2w)	Persistent, recurrent, or metastatic CC; progression after at least one prior systemic chemotherapy regimen; squamous cell carcinoma, adenosquamous carcinoma, or adenocarcinoma; ECOG performance status 0 or 1, measurable disease per RECIST 1.1 criteria	Lower anti-tumor activity, but with good safety and high clinical tolerance	[192]
		Nivolumab in combination with concurrent chemoradiotherapy (CRT)	NCT03298893	I/II	Nivolumab (240 mg flat dose q2w) plus concurrent cisplatin (40 mg/m^2^ weekly) and definitive radiotherapy (including image-guided brachytherapy)	16 treatment-naïve women with locally-advanced cervical cancer (LACC; FIGO 2018 stages IB3–IVA)	ORR was 93.8%; the 2-year PFS rate was 75%, showing good anti-tumor activity and durable clinical efficacy	[193]
	HER2	Trastuzumab Deruxtecan (T-DXd)	NCT04482309	II	5.4 mg/kg administered intravenously once every 3 weeks (21-day cycle)	Histologically confirmed locally advanced, unresectable, or metastatic cervical carcinoma; ECOG performance status 0–1; progressed after ≥1 prior systemic therapy or had no satisfactory alternative treatment options	ORR of 50% in heavily pretreated HER2-expressing CC, better efficacy was observed in IHC 3+ patients (ORR 75%), with a median PFS of 7.0 months	[194]
	HER2(ERBB2)	Neratinib	NCT01953926	II	240 mg/day PO QD continuously; loperamide prophylaxis was mandatory during cycle 1	HER2-mutant, metastatic or recurrent CC, progressing after platinum-based treatment	The overall response rate (ORR) was 18.2%, the clinical benefit rate (CBR) was 45.5%, the duration of response (DoR) was 7.6 months, and the progression-free survival (PFS) was 5.1 months, showing a moderate anti-tumor activity and relatively limited durable benefits	[195]
EC	ErbB family	Afatinib + Carboplatin + Paclitaxel	NCT00809133	I	Afatinib 20–40 mg/day PO; IV D1, q21d	Advanced solid tumors (including EC) R/M EC pMMR R/M EC	The ORR of the A/C regimen is 25%, and the RP2D of the A/C/P regimen is A 20mg + C AUC5 + P 175 mg/m^2^ with an ORR of 19%. The addition of Paclitaxel did not significantly improve the anti-tumor efficacy	[196]
	Ang1/2	Trebananib (AMG386)	NCT01210222	II	15 mg/kg IV QW (every week); one cycle defined as 28 days	P/R EC, measurable disease, and 1–2 prior chemotherapy lines	Insufficient activity, further research is not recommended	[197]
	PD-1/VEGFR1-3/FGFR1-4/EGFR/PDGFR/c-Met	Sintilimab + Anlotinib	NCT04157491	I/II	Sintilimab 200 mg was administered intravenously on day 1 every 3 weeks, and anlotinib 12 mg (orally) was administered on days 1–14 in a 21-day cycle	Recurrent or advanced EC who had progressed on or after at least one line of standard platinum-based chemotherapy	Potent and long-lasting anti-tumor activity	[198]
	PD-1	Pembrolizumab + Paclitaxel + Carboplatin	NCT03914612	III	Pembrolizumab 200 mg IV q3w for 6 cycles, followed by 400 mg IV q6w for up to 14 maintenance cycles; plus Paclitaxel 175 mg/m^2^ and Carboplatin AUC 5 IV q3w	Measurable disease (stage III or IVA) or stage IVB or recurrent EC, stratified by dMMR or pMMR status	Significantly prolonged the PFS of patients with advanced or recurrent diseases	[199]
		Pembrolizumab	NCT04634877	III	Pembrolizumab 200 mg IV Q3W for 6 cycles with carboplatin-paclitaxel, followed by pembrolizumab 400 mg IV Q6W for 6 cycles	Newly diagnosed, high-risk EC (FIGO stage I/II non-endometrioid or p53-aberrant, or stage III/IVA) with no evidence of disease post-surgery	It did not improve the disease-free survival of the entire population, but showed clinically meaningful benefits in the dMMR subgroup	[200]
	VEGFR1-3/FGFR1-4/PDGFRα/RET/KIT/PD-1	Lenvatinib + Pembrolizumab	NCT02501096	I/II	Lenvatinib 20 mg/day PO; Pembrolizumab 200 mg IV q3w	Advanced EC Patients, progressed after approved therapies	Good anti-tumor activity	[201]
	mTOR	Vistusertib + Anastrozole vs. Anastrozole	NCT02730923	I/II	V+A arm:oral vistusertib (125 mg twice daily on 2 days per week) in combination with oral anastrozole (1 mg daily) A arm: oral anastrozole alone	HR^+^ (ER and/or PR ≥ 1%), recurrent or metastatic EC	Combination therapy can improve PFS and ORR in patients with hormone receptor-positive recurrent/metastatic disease	[202]
	PI3K	BKM120 (buparlisib)	NCT01397877	II	100 mg PO qd, then reduced to 60 mg PO qd	Advanced or recurrent EC, ≤1 prior chemotherapy regimen	The median PFS in the 60 mg group was 4.5 months, with no objective response	[203]

Abbreviations: AUC: area under the curve; Ang: angiopoietin; CBR: clinical benefit rate; CCL2: CC-chemokine ligand 2; CC: cervical cancer; CD14dimCD16+: CD14dim CD16bright; CSF1R: colony-stimulating factor-1 receptor; CT: chemotherapy; DoR: duration of response; dMMR: deficient mismatch repair; EC: endometrial cancer; ECOG: Eastern Cooperative Oncology Group; EGFR: epidermal growth factor receptor; ER: estrogen receptor; FGFR: fibroblast growth factor receptor; FIGO: International Federation of Gynecology and Obstetrics; GOG: Gynecologic Oncology Group; HER2: human epidermal growth factor receptor 2; HR: hormone receptor; IL-12: interleukin-12; IP: intraperitoneal; ITT: intention-to-treat; IV: intravenous infusion; LACC: locally-advanced cervical cancer; M: metastatic; mTORC: mammalian target of rapamycin complex; OC: ovarian cancer; ORR: objective response rate; OS: overall survival; P: persistent; PDGFR: platelet-derived growth factor receptor; PD-L1: programmed death-ligand 1; pMMR: proficient mismatch repair; PLD: pegylated liposomal doxorubicin; PO: per os (by mouth); PR: progesterone receptor; PS: performance status; PFS: progression-free survival; qd: quaque die (once daily); qw: weekly; R: recurrent; TAMs: tumor-associated macrophages; VEGF: vascular endothelial growth factor; VEGFR: vascular endothelial growth factor receptor.

CSF1R inhibitors: Given the central role of CSF1–CSF1R signaling in TAM survival across OC, CC, and EC, pharmacologic blockade of this axis has emerged as a primary therapeutic strategy that follows a logical progression from mechanism validation to clinical testing. Preclinical investigations provide support for this therapeutic strategy in OC. PLX3397 combined with paclitaxel inhibits tumor growth and prolongs survival by promoting M2-like TAM reprogramming toward M1-like phenotypes and enhancing antitumor immune responses. This combination regimen demonstrates favorable safety in vivo, providing a potential targeted strategy for OC treatment [204]. Beyond OC, CSF1R inhibitors also demonstrate unique value in CC treatment research. In CC preclinical studies, the CSF1R inhibitor BLZ945 exhibits significant single-agent antitumor activity, achieving tumor stasis in keratin 14-human papillomavirus type 16 (K14-HPV-16) transgenic mouse models. This effect surpasses its performance in breast cancer models, where it merely delayed tumor growth [205]. This suggests that BLZ945’s mechanism of action in the CC microenvironment may differ from other tissue types.

Similarly, targeting CSF1R signaling, PLX3397 demonstrates selective inhibitory effects in EC research. EC cells secrete CSF1 to promote TAM migration, while the CSF1R inhibitor PLX3397 blocks this process and selectively inhibits EC cell proliferation without affecting normal endometrial cells. Additionally, PLX3397 downregulates proliferation-related signaling molecules, including JAK-1 and AKT [206]. Early translational research using advanced experimental models further elucidates CSF1R inhibitor mechanisms. Three-dimensional microfluidic model studies demonstrate that the CSF1R inhibitor BLZ945 reduces TAM infiltration. The platinum-based chemotherapeutic agent cisplatin induces increased CSF1 secretion from cancer cells, enhancing TAM recruitment. Concurrent CSF1R blockade is associated with reversal of this effect, suggesting synergistic potential for this strategy combined with chemotherapy [207]. Prospective clinical evidence validates these laboratory observations. As an oral small-molecule CSF1R inhibitor, pexidartinib inhibits monocyte recruitment and differentiation into the TME by blocking CSF1R signaling. In a phase I trial of platinum-resistant or refractory OC/peritoneal cancer, pexidartinib combined with paclitaxel achieved remission in two out of six patients and significantly reduced the proportion of CD14dim/CD16^+^ monocytes [208]. These results suggest an association between pexidartinib treatment and attenuated pro-tumoral TAM activity, potentially linked to CSF1R signal modulation, thereby supporting the rationale for TAM-targeted therapeutic strategies in OC.

CCR2 inhibitors: While CSF1R inhibition targets TAM survival, complementary blockade of monocyte recruitment via the CCL2–CCR2 axis offers a distinct and synergistic approach grounded in the same mechanistic framework. CCR2 inhibitors demonstrate significant potential in treating gynecologic malignancies by blocking CCL2-mediated TAM recruitment. At the preclinical level, CCL2 promotes tumor progression by activating the MEK/ERK/MAP3K19 signaling pathway via CCR2. Existing research suggests that the CCL2–CCR2 axis may participate in immunosuppression by regulating TAM recruitment, and the CCR2 inhibitor RS504393 can effectively block this effect [209]. On this basis, bromodomain and extra-terminal domain inhibitors correlates with reduced CCR2 expression in TAMs, M2-like TAM apoptosis, and potential reprogramming into M1-like TAMs. The combination of this inhibitor with bevacizumab is associated with reduced TAM infiltration, potential reversal of the resistant microenvironment, and superior safety compared to CSF1–CSF1R inhibitors [210]. These strategies targeting the CCL2–CCR2 axis have also been validated in CC. In CC, the CCR2 antagonist RS504393 significantly inhibits tumor cell migration, invasion, and in vivo tumor growth. Combined use with CMTM6 knockdown produces synergistic antitumor effects. Additionally, TAM clearance mediated by anti-F4/80 antibodies significantly inhibits tumor progression [141]. This confirms that dual strategies blocking TAM recruitment and survival possess clinical value. In EC models, genetic knockout of the CCL2 gene prolongs median survival in mice, reduces tumor burden, and reverses peripheral blood monocytosis. Retrospective biomarker analyses offer complementary clinical evidence. In EC research, LKB1 functions as a serine/threonine kinase. Analysis of 175 primary EC tissue microarrays demonstrates that LKB1 protein levels are significantly negatively correlated with CCL2 expression and CD68^+^ TAM density [211]. Prospective trial evidence and approved clinical applications specifically targeting the CCL2–CCR2 axis in gynecologic malignancies have not yet been realized. These results indicate that CCR2 inhibitors or CCL2-neutralizing antibodies may inhibit EC progression by blocking monocyte mobilization from bone marrow to blood and their subsequent recruitment to tumors.

Bisphosphonates: Bisphosphonates demonstrate antitumor activity in treating gynecologic malignancies by depleting TAMs and inhibiting angiogenesis. Preclinical studies demonstrate that clodronate reduces tumor weight in orthotopic models of OC and blocks angiogenesis by depleting TAMs and inhibiting endothelial cell function. This drug decreases TAM density and microvessel density and inhibits the secretion of multiple pro-angiogenic cytokines [212]. Zoledronic acid (ZA) eliminates tumor growth-promoting effects in chronic-stress-induced OC models, blocks TAM infiltration and platelet-derived growth factor AA (PDGF-AA) expression, and reduces the number of intratumoral CD68^+^ TAMs, supporting its potential value for OC patients with psychological stress [213]. In CC models, ZA inhibits the expression and activation of matrix metalloproteinase-9 (MMP-9) in TAMs and blocks VEGF signal transduction to inhibit angiogenesis. In HPV16 transgenic mouse models, ZA reduces the incidence of progression from precancerous lesions to invasive cancer and shrinks the volume of established tumors [214]. This drug has a well-established clinical safety profile, but its anti-TAM efficacy in human CC remains to be validated. Beyond direct TAM depletion, combinations of bisphosphonates and immunomodulators also demonstrate synergistic potential. In HPV16 E6/E7^+^TC-1 models, clodronate liposomes effectively eliminate TAMs and increase the M1-like/M2-like TAM ratio. Combined application with TLR2 agonist-conjugated peptides significantly inhibits tumor growth and prolongs survival [215]. Extending into early translational investigations, studies in EC demonstrate that depleting TAMs through local injection of macrophage-depleting liposomal clodronate enhances the antitumor effects of radiotherapy [150]. CSF1R inhibitors, CCR2 antagonists, and bisphosphonates have demonstrated potential for modulating TAMs in preclinical studies of OC, CC, and EC. However, these studies are largely limited to mouse models or cell experiments. Therefore, large-scale clinical trials are urgently needed to validate the survival benefits of these strategies in humans and clarify the feasibility of their combination with chemotherapy or immunotherapy.

Monotherapies targeting TAMs have demonstrated limited antitumor efficacy in clinical trials. Although agents such as emactuzumab and carlumab effectively reduce TAM abundance or suppress monocyte infiltration, they have failed to deliver meaningful clinical benefits. These findings underscore the need to consider complex compensatory cellular networks within the TME, particularly the interdependence between TAMs and stromal constituents, including CAFs and mesothelial cells. In OC, paclitaxel-based chemotherapy increases the proportion of M2-like TAMs, whereas combined CSF-1R inhibition and chemotherapy concurrently reduce M2-like TAMs, MDSCs, neutrophils, and inflammatory monocytes while increasing CD8^+^ T cell frequencies [204]. In CC, single-cell and spatial transcriptomics have identified CD54^+^ iCAFs and ITGAL^+^ macrophages forming immunosuppressive niches at the tumor–stroma interface. CD54^+^ iCAFs polarize macrophages toward an M2-like phenotype via CCL2 secretion and CD54–ITGAL direct contact, which triggers macrophage autocrine CXCL8 and upregulates PD-L1, impairing CD8^+^ T cell function [216]. In EC, CAFs secrete CCL2, IL-6, and TGF-β, among other mediators, to promote monocyte polarization toward M2-like TAMs [217]. These network dependencies suggest that future clinical trials should prioritize multi-target combination regimens that integrate TAM depletion with CAF-directed or mesothelial cell modulation, rather than continuing to pursue single-population depletion strategies.

#### 4.1.2. Reprogramming TAMs

Beyond depleting or blocking TAM recruitment, an alternative paradigm seeks to functionally reprogram existing TAMs from pro-tumoral M2-like to anti-tumoral M1-like phenotypes. This approach leverages the metabolic and signaling plasticity described above and has been advanced through targeted delivery systems and pathway modulation. Reprogramming M2-like TAMs with pro-tumoral phenotypes into antitumor M1-like phenotypes is being pursued in immunotherapy for gynecologic malignancies. Currently, multiple reprogramming agents and technical approaches have been developed, including targeted chimeric molecules, conditionally activated immune cytokines, and metabolic regulators. These strategies are complementary and synergistic, progressively refining the therapeutic framework for TAM reprogramming and providing novel research directions for clinical translation and protocol optimization for precision immunotherapy in gynecologic tumors.

Nanoparticles (NPs): Technological advances in nanomedicine have established that NPs possess outstanding targeting capabilities, high drug delivery efficiency, and favorable biocompatibility, enabling TAM recognition and modulation of functional status. Application of these technologies in gynecologic malignancies including OC, CC, and EC demonstrates that they provide innovative therapeutic avenues. Preclinical investigations in OC demonstrate foundational targeting capabilities, as folate-targeted nanoparticles (FA-DCNPs) can precisely target M2-like TAMs through folate receptor 2 (FOLR2), delivering the glutamine metabolism inhibitor DON to block M2-like polarization and promote M1-like conversion. This has been validated in OC ID8 models, where FA-DCNPs significantly increased the proportion of M1-like TAMs in tumor tissues [218]. Beyond small-molecule drug delivery, glycosylated nano-platforms also hold significant application value in TAM-targeted reprogramming. Dimannose-modified mRNA NPs target M2-like TAMs through CD206, delivering mRNA encoding IRF5 and its activating kinase IKKβ to achieve genetic reprogramming. In OC peritoneal dissemination models, this strategy achieved complete tumor regression in 40% of cases, prolonged median survival, and significantly reduced the proportion of M2-like macrophages while increasing M1-like macrophage proportions [219].

Extending into CC applications, NP-mediated TAM reprogramming strategies have also achieved important advances. Nanoemulsions loaded with Toll-like receptor 7/8 agonists can activate antigen-presenting cells, inducing the transformation of M2-like TAMs toward M1-like phenotypes [220]. Polyethyleneimine-modified dendritic mesoporous silica NPs loaded with microRNA-125a can drive TAM polarization toward M1-like phenotypes. In C57BL/6J mouse TC-1 CC models, this treatment significantly downregulated M2-like marker expression and promoted CD8^+^ T cell infiltration [221]. These preclinical observations suggest potential applicability in hormone-dependent malignancies. Beyond OC, NP-mediated TAM reprogramming demonstrates potential in treating other hormone-dependent cancers. Yoon and colleagues constructed a biomimetic nano-platform that effectively induced M2-like TAM transformation toward M1-like phenotypes and upregulated the expression of proinflammatory markers including inducible iNOS through the upconversion of NP-mediated photodynamic therapy combined with oxygen delivery and chemotherapy [222]. This strategy significantly improved the tumor hypoxic microenvironment and enhanced the antitumor efficacy in 4T1 breast cancer models. Given that EC includes hormone-dependent tumors, this targeted strategy holds potential application value in EC.

PI3K inhibitors: Complementing nanoparticle-based delivery, small-molecule pathway inhibition offers a pharmacologically distinct approach to TAM reprogramming. The PI3K/AKT pathway represents a key node for this intervention. Evidence from preclinical studies across multiple cancer types indicates that PI3K inhibitors exhibit potential therapeutic applications by modulating TAMs’ phenotype and functional status. In the context of gynecological malignancies, including OC, CC, and EC, this mechanism has been validated. In OC research, multiple studies have confirmed that PI3K pathway inhibition can regulate TAM polarization direction and exert antitumor effects. Gallic acid (GA) downregulates CD47 expression via PI3K/AKT pathway inhibition, promoting TAM polarization toward the M1-like phenotype and enhancing their phagocytic activity. This effect has been validated in co-culture systems of A2780 OC cells with THP-1 macrophages and in ID8 OC tumor-bearing mouse models [223]. Triptolide (TPL) blocks M2-like TAM polarization through PI3K/AKT/NF-κB pathway inhibition, and this mechanism has been demonstrated in A2780/DDP cisplatin-resistant tumor cells and OC xenograft nude mouse models [224].

The regulatory effects of PI3K inhibitors on TAMs in CC have also been demonstrated. In CC, M2-like TAMs can activate the PI3K/AKT signaling pathway, upregulating PD-L1 expression in SiHa and HeLa CC cells, and the PI3K inhibitor LY294002 can partially block this regulatory process [225]. PI3K inhibitors demonstrate similar potential therapeutic prospects in EC treatment. The PI3K/AKT signaling pathway is activated in TAMs and closely associated with the function of long non-coding RNA GAS5. GAS5 adsorbs microRNA-21 through a competitive mechanism, mitigating its inhibitory effect on PTEN and suppressing the PI3K/AKT pathway. PTEN overexpression can upregulate the M1-like marker CD86 and inhibit the M2-like marker CD206 [226]. These findings suggest that PI3K/AKT pathway inhibition may contribute to TAM polarization regulation in EC, providing a basis for TAM reprogramming strategies.

M1 macrophage-engineered vesicles (M1MEVs): M1MEVs represent an innovative immunotherapeutic strategy. Generated through nitrogen cavitation, this technique produces abundant nanoscale vesicles derived from M1-like macrophages. Initially explored in osteosarcoma (OS), researchers developed empty vesicles (E-MVs) and cisplatin-loaded vesicles (C-MVs), with both demonstrating substantial immunomodulatory and antitumor activities. In the 143B orthotopic OS xenograft model, E-MV and C-MV treatments significantly increased the intratumoral ratio of M1-like to M2-like TAMs, accompanied by markedly elevated serum TNF-α levels. Compared with free cisplatin, C-MVs displayed lower IC50 values against OS cells in vitro and significantly extended median survival in vivo. Moreover, C-MVs avoided the body weight loss and hepatorenal toxicity associated with equivalent doses of free cisplatin, demonstrating favorable safety profiles [227]. This strategy was subsequently applied to OC, where M1MEVs prepared from human peripheral blood mononuclear cell (PBMC)-derived M1-like macrophages effectively encapsulated therapeutic cargoes. In vitro experiments demonstrated that M1MEVs efficiently repolarized M2-like TAMs toward M1-like TAMs, evidenced by markedly elevated TNF-α secretion and upregulated CXCL8 mRNA expression. In OC cell co-culture systems, M1MEV treatment significantly enhanced TNF-α production and exhibited dose-dependent growth inhibition of cancer cells, with tumor-localizing capacity demonstrated in mouse models [228]. Given that CC and EC also harbor immunosuppressive microenvironments dominated by M2-like TAMs, application of this technology in these malignancies is theoretically viable. However, specific efficacy and safety profiles require validation through preclinical investigations.

Currently, therapeutic TAM reprogramming strategies targeting OC, CC, and EC have been established through a comprehensive multi-directional research framework. Both NP-mediated targeted delivery and PI3K pathway inhibition promote the transition of M2-like TAMs toward the M1-like phenotype, demonstrating promising prospects in enhancing antitumor immunity and suppressing tumor progression. Additionally, M1MEVs provide an innovative vesicle-based approach that efficiently repolarizes M2-like TAMs toward the M1-like TAMs while demonstrating favorable tumor-targeting capacity and safety profiles in preclinical models. Beyond these biochemical and vesicle-based approaches, emerging physical interventions also demonstrate complementary TME-modulating capabilities with immunotherapy potential. Exploratory studies in hepatocellular carcinoma-bearing mouse models provide preclinical evidence that synergistic pulsed electric fields (PEFs) remodel the TME by reducing extracellular matrix stiffness and density and alleviating hypoxia, physicochemical barriers that are also implicated in gynecologic malignancies. These changes facilitate intratumoral infiltration of endogenous cytotoxic immune cells, including CD8^+^ T cells and NK cells, and significantly enhance enrichment and parenchymal penetration of exogenous NK cells within tumors [229]. These findings provide a preliminary experimental basis for clinical translation studies combining PEF with cellular immunotherapy, offering a non-pharmacological approach to overcome physical barriers in the TME of OC, CC, and EC. However, current research remains largely confined to cellular and animal models, and specific variations in reprogramming strategies across different organs and tumor subtypes, together with long-term safety profiles, require further clarification. Future efforts should advance additional preclinical translational studies and clinical trials to establish optimal application protocols and combination strategies for TAM reprogramming across various types of gynecological malignancies.

#### 4.1.3. Combined Immunotherapy Strategies: PD-1 Inhibitors

The preclinical efficacy of TAM-targeted monotherapies remains limited by compensatory mechanisms within the TME. This has driven the development of combination strategies pairing TAM modulation with immune checkpoint inhibition. Preclinical investigations have established foundational evidence for combination strategies targeting TAMs and PD-1 inhibitors across gynecological cancers. In OC, combined application of the C5aR1 inhibitor PMX53 with PD-1 inhibitors is linked with TAM polarization toward M1-like TAMs and enhanced CD8^+^ T cell function, which may contribute to overcoming the limitations of monotherapy [230]. The PI3Kγ inhibitor IPI-549 reprograms TAMs into an immune-activated phenotype through PI3Kγ–AKT–NF-κB pathway inhibition, and combined treatment with PD-1 inhibitors is associated with suppressed tumor growth and prolonged survival in preclinical models [231]. Preclinical studies have also provided important mechanistic support for TAM-targeted therapy combined with PD-1 inhibitors in CC treatment. Combined application of TGF-β inhibitors or CSF-1R inhibitors with PD-1/PD-L1 inhibitors enhances antitumor efficacy, providing a theoretical foundation for improving therapeutic outcomes and combination strategies in CC patients [225]. Translational studies using human specimens have extended these findings into clinical relevance.

In EC, the CSF1R inhibitor FF-10101 combined with PD-1 inhibitors reduces immunosuppressive TAMs expressing PD-L1 and PD-L2 through sustained CSF1R signal inhibition and promotes M1-like TAM polarization, demonstrating antitumor effects superior to either monotherapy. In human EC specimens, FF-10101 similarly decreases the proportion of PD-L1+ TAMs and upregulates M1-like TAM-related gene expression [37]. In CC, TAMs serve as critical mediators for the efficacy of neoadjuvant chemotherapy (NACT) combined with PD-1 blockade therapy. Following NACT treatment, interactions between macrophages and cancer cells are enhanced, while CD74 expression is upregulated in TAMs and restricts therapeutic efficacy by inhibiting phagocytic function and promoting M2-like polarization. Tumors exposed to CD74 blockade in combination therapy exhibited enhanced antitumor activity [121]. Retrospective biomarker analyses have identified immune checkpoint expression profiles predictive of clinical outcomes. In locally advanced CC treated with concurrent chemoradiotherapy (CRT) combined with PD-1 inhibitors, TAMs’ immune checkpoint expression profile correlates with clinical outcomes. TAMs from progression-free (PF) patients highly express inducible co-stimulator ligand (ICOS-L), whereas TAMs from progressive disease (PD) patients highly express PD-L1 [193]. In EC, the phase III trial (NCT03517449) conducted by Makker et al. confirmed that lenvatinib combined with pembrolizumab significantly improves median progression-free survival and median overall survival compared with chemotherapy in patients with pMMR EC. These prospective findings have achieved approved clinical relevance. The lenvatinib–pembrolizumab regimen is now an established standard-of-care treatment for patients with advanced or recurrent pMMR endometrial cancer, representing successful clinical translation of TAM-targeted combination immunotherapy in gynecological malignancies [232]. Nonetheless, the combination regimen has a high incidence of high-grade adverse events, with some patients stopping treatment due to toxic reactions, necessitating safety monitoring in clinical use.

The therapeutic strategy of targeting TAMs with PD-1 inhibitors is commonly used to overcome immune therapeutic resistance targeting TAM polarization into M1-like phenotypes in gynecological malignancies. However, clinical translation of this combination faces limitations due to the number of high-grade adverse events and the low predictability of therapeutic effectiveness given TAMs’ phenotypic heterogeneity. Future studies should target selective goal optimization, perform cross-tumor efficacy prediction model development, and identify optimal integration patterns with existing treatment modalities to enable standardized implementation for gynecological malignancies.

### 4.2. TAMs’ Prognostic Biomarkers and the Emerging Potential for Precision Medicine in Gynecological Tumors

Recent advancements in single-cell sequencing, multiplex immuno-fluorescence, and artificial-intelligence-aided analysis technology have facilitated the clinical translational application of TAM-based prognostic prediction models in OC, CC, and EC. Models with greater prognostic power than classical clinicopathological parameters may enable more accurate patient stratification and risk assessment.

Beyond single-omics analysis, multi-omics integration via network-based computational methods offers enhanced resolution for heterogeneous biomarker discovery. Researchers validated this finding using FUNMarker. The approach first clusters subpopulations based on gene expression data to reduce sample heterogeneity and subsequently integrates seven categories of protein–protein interaction networks into a unified fusion network, thereby addressing the incompleteness inherent to single-network sources. A label propagation algorithm is then applied to rank and identify subpopulation-specific biomarkers. FUNMarker demonstrated superior discriminatory capacity in stratifying patients with distinct prognostic outcomes and effectively captured intratumoral molecular heterogeneity [233]. This approach exemplifies how computational integration of diverse data modalities can dissect tumor heterogeneity, a strategy readily applicable to integrating TAMs’ transcriptomic, proteomic, and spatial data in gynecological cancers. Complementing network-based methodologies, artificial intelligence approaches provide alternative computational frameworks for TAM detection. The use of AI technology improves the predictive power of TAM markers. Through NaroNet, a weakly supervised deep learning strategy for analyzing multiplex immunofluorescence images, one can identify the local phenotype P1 enriched with CD68^+^ TAMs. The cellular neighborhood N8 can also be identified using NaroNet. These characteristics are associated with recurrence-free tumors and provide better predictive performance than models based on mismatch repair protein status and POLE mutation status [234].

By translating these computational frameworks into experimental platforms, single-cell sequencing technologies enable the development of clinically applicable risk stratification tools. Researchers have developed a TAM-related gene risk score model through single-cell sequencing technology. They confirmed that higher risk scores are associated with poorer prognosis. The team also determined that high-risk patients respond better to immunotherapy [235]. Advances in multiplex imaging modalities further facilitate the dynamic monitoring of therapeutic responses. Multiplex immunofluorescence technology, for instance, has been used to dynamically evaluate changes in the TME before and after neoadjuvant chemotherapy. The results showed that decreased M2-like TAMs infiltrated in chemotherapy, and elevated M1-like/M2-like ratios were associated with a positive treatment response, providing a foundation for dynamic monitoring and the application of TAM markers during treatment [236].

Application of these integrated technologies in large-scale clinical cohorts validates the prognostic significance of TAM signatures. For instance, the researchers’ transcriptomic data from single cells and bulk cells to generate 219 genes related to TAMs through weighted gene co-expression network analysis, the basis of a six-gene prognostic signature. This signature outperformed the classical clinicopathological characteristics in predicting the OS and proved to be a better tool for reliable stratification in large-scale clinical samples [237]. In the context of CC prognostic assessment, the clinical relevance of TAMs is strong. High-density M2-like TAM (CD163^+^CD204^+^) activates the STAT3/NF-κB signaling pathway. Its anticancer immunotherapy effectiveness via the establishment of an immunosuppressive loop leads to poor clinical outcomes. The combination of M2-like TAMs and the STAT3/NF-κB pathway effect may be a promising predictive marker for CC prognosis according to these results [80]. CD163 is superior to CD68 in predicting tumor-associated macrophage activity. According to the findings of a study of 130 samples of cervical tissue, CD163^+^ TAM counts correlate better with CC malignant transformation, which is associated with greater clinical value malignant transformation and metastasis [238]. In addition, the relative balance of M1-like and M2-like TAMs should be considered for prognostic value. High expression levels of CD11c, a marker for M1-like TAMs, correlate significantly with prolonged OS. Combined assessment of infiltration abundance of M1-like TAMs with expression levels of CD24 and CD47 demonstrates improved prognostic risk stratification ability over each marker alone [239]. The importance of TAMs in prognostic EC prediction has also been confirmed. Multiplex quantitative immunofluorescence technology was used to recognize five immune phenotypes for low-grade early-stage EC. The macrophage-enriched phenotype had high CD68^+^ TAMs expression, whereas the immune-excluded phenotype was characterized by increased expression of PD-L1 on the surface of TAMs and contributes to tumor relapse. The predictive model created from integrated immune phenotypes outperformed traditional models built on classical pathological variables [240]. Imaging mass cytometry analysis of 40 EC patients further revealed the predictive interactive signal of CXCR4-high M2-like TAMs and CD90^+^CD105^+^ endothelial cells, together with Tregs, for adverse outcomes. Models using cell frequency cellular interaction and functional marker expression built using random forest can also predict recurrence risk in a copy-number-high subtype [241].

These TAM-marker TAMs demonstrate established prognostic value, while their utility as predictive biomarkers for specific therapeutic selection remains theoretical and awaits prospective clinical validation. Elevated proportions of CD14dim/CD16^+^ monocytes in platinum-resistant patients are associated with active CSF1R signaling, providing a preclinical rationale for investigating CSF1R inhibition strategies, though clinical predictive utility remains unvalidated. High-density infiltration of M2-like TAMs (CD163^+^CD204^+^) or enrichment of CD74^+^ TAMs indicates an immunosuppressive TME that renders CSF1R inhibitors or CD74-targeted blockade combined with PD-1 inhibitors potentially appropriate therapeutic options. Increased proportions of PD-L1^+^ TAMs are associated with a predominance of immune-checkpoint-mediated immunosuppressive mechanisms, suggesting hypothetical responsiveness to combined CSF1R inhibition and PD-1 blockade that requires confirmation in clinical trials. It is also critical to distinguish between prognostic association and predictive utility. Most current TAM biomarker studies in gynecological malignancies are retrospective, single-arm, or preclinical, lacking the randomized comparative data necessary to validate treatment selection algorithms. Achieving this translational goal will ultimately empower clinicians to administer personalized biomarker-guided TAM-targeted therapies, thereby advancing precision medicine paradigms for gynecological malignancies.

### 4.3. Cancer-Specific Clinical Application of TAM Biomarkers

TAM markers demonstrate significant tumor-specific clinical value in OC, CC, and EC, with their expression profile variations closely associated with the distinct TME characteristics and prognostic stratification requirements of each malignancy. In OC, the proportion of circulating intermediate blood monocytes (IBMs) demonstrates significant positive correlation with the immunosuppressive phenotype of ascitic TAMs (CCR2high/CD163high/CD206high/CD86low) and the peritoneal carcinomatosis index, reflecting the immunosuppressive microenvironment of ascites and tumor burden. Combined detection of IBM and TAM markers can guide the assessment of immunotherapy response and the adjustment of treatment decisions in OC [242]. High CD163 expression on TAMs in ascites is associated with early recurrence following first-line HSCG therapy, whereas levels of CD204 and von Willebrand factor derived from TAMs in plasma can effectively predict patient recurrence-free survival [243]. Collectively, these findings demonstrate that TAM-related markers can serve as quantitative indicators of immunosuppressive burden in the peritoneal microenvironment. TAM markers also demonstrate prognostic predictive value associated with HPV status in CC. Multiplex immunofluorescence detection reveals that the density of CD68^+^CD163^+^M2-like TAMs in the TME of HPV^+^CC patients is significantly higher than in HPV-negative patients, and this marker possesses prognostic value in CC patients, with high M2-like TAM density associated with increased risk of disease progression [244].

Extending to the EC domain, TAMs can serve as important markers for guiding molecular subtyping-based treatment. Multiplex immunofluorescence analysis of the TME across different molecular subtypes of EC reveals increased M2-like TAM infiltration and the highest proportion of PD-L1^+^TAMs in the TP53-mutant subtype, indicating its immunosuppressive TME characteristics [98]. Regarding the peritoneal dissemination characteristic of OC, the association between HPV status and M2-like TAM infiltration in CC, and the molecular subtyping framework of EC, although TAM markers exhibit differentiated application patterns across these three malignancies, their core essence converges upon the quantification of TME immunosuppressive burden as a shared biological principle. Future research should establish cross-tumor comparable TAM functional stratification standards while maintaining tumor-specific characteristics, thereby providing a unified methodological framework for TME-directed individualized immunotherapy.

### 4.4. Optimized Toxicity Management and Precision Stratification Strategies

Combination regimens targeting TAMs and PD-1 inhibitors demonstrate elevated rates of high-grade adverse events, necessitating refined toxicity management protocols to address these challenges. Combinations such as LY3022855 targeting the CSF1R pathway plus durvalumab remain generally tolerable yet exhibit limited clinical activity [176]. This restricted clinical benefit may relate to non-selective macrophage depletion or secondary accumulation of alternative pro-tumorigenic immune cells including tumor-associated neutrophils [245]. Patients receiving ICIs who develop severe immune-related adverse events (irAEs) display inflammatory profiles dominated by M2-like TAMs, which comprise over 50% of immune cells in affected organs such as the pituitary and adrenal glands. This pathological mechanism suggests that systemic macrophage depletion may disrupt the immunosuppressive functions of M2-like TAMs and trigger uncontrolled inflammatory responses, subsequently resulting in multi-organ toxicity [246]. Consequently, management strategies may employ staggered dosing schedules coupled with dynamic monitoring of circulating biomarkers. CD40 agonists enhance antigen-presenting cell function and synergize with ICIs, although systemic macrophage activation may precipitate treatment-related adverse events [247]. Current management approaches increasingly favor conditionally active bispecific antibodies or intratumoral delivery of oncolytic viruses bearing CD40 ligand genes, restricting immune activation to the local TME. The combination of the PI3Kγ inhibitor eganelisib with nivolumab selectively suppresses the PI3Kγ isoform enriched in macrophages, enabling the reprogramming of immunosuppressive cells while minimizing off-target risks associated with broad pathway inhibition [248]. Furthermore, anti-TREM2 monoclonal antibodies combined with pembrolizumab have entered clinical trials, with toxicity management relying on the selective depletion of M2-like TAMs via antibody-dependent cellular cytotoxicity [249]. These modality-specific refinements illustrate the field’s evolution from systemic immunomodulation toward precision-targeted interventions, with future optimization requiring the integration of predictive biomarkers to refine patient stratification criteria. Stratification via the Immunotherapy Response Score (IRS) in gynecological malignancies offers an important paradigm. Among these tumors lacking approved PD-1 monotherapy indications, only approximately 11.7% of patients exhibit high-risk IRS status, with 7.6% representing a subset characterized by high-risk IRS yet low-risk tumor mutational burden. This stratification approach enables the precise identification of patients likely to benefit from PD-1 monotherapy, preventing ineffective treatment in low-risk IRS cohorts while avoiding unnecessary combination chemotherapy toxicities in high-risk IRS patients [250]. Integration of such biomarker-driven stratification strategies with therapeutic modalities selectively depleting M2-like TAMs may maximize anti-tumor immune activation while minimizing high-grade adverse event risks, ultimately realizing the clinical translational value of combination strategies in gynecological malignancies.

## 5. Future Perspectives

### 5.1. Chimeric Antigen Receptor Macrophage (CAR-Ms) Technology

CAR-Ms therapy has emerged as a novel immune cell therapeutic strategy demonstrating unique advantages in solid tumor treatment. CAR-Ms technology leverages macrophage penetration capabilities into solid tumors and their phagocytic functions to overcome the limitations of chimeric antigen receptor T cells (CAR-T cells) in solid tumor therapy. Through genetic engineering modification, CAR-Ms can be induced to maintain the M1-like phenotype, thereby equipping CAR-Ms with antigen-specific phagocytic and tumor clearance capabilities [251]. CAR-Ms technology can enhance antitumor immune responses through TME remodeling. Clinical trials at the pan-cancer level have preliminarily validated the feasibility of CAR-Ms therapy for solid tumors. CT-0508 (NCT04660929) treated 14 patients with advanced HER2-positive solid tumors, among whom 4 out of 9 patients with HER2 immunohistochemistry (IHC) 3^+^ achieved disease stabilization after 8 weeks of treatment, whereas 5 patients with HER2 IHC 2^+^ showed no therapeutic response [252]. These results suggest that antigen expression levels may influence the clinical efficacy of CAR-Ms and provide important references for subsequent research. In gynecological malignancies, research on CAR-M therapy has achieved significant progress. For OC, researchers designed CD47-targeting CAR-Ms with the co-expression of IL-21 through P2A linker. IL-21-modified CAR-Ms can upregulate M1-like polarization marker CD86 and enhance antigen presentation capabilities [253]. In preclinical studies, HER2-targeting and CD47-targeting CAR-Ms demonstrated antigen-dependent phagocytic capabilities against OC cell lines SKOV3 and A2780. CAR-Ms treatment can increase the M1-like/M2-like macrophage ratio in tumor tissues, reshaping the phenotypic balance of TAMs in the TME [254]. In contrast, research on CAR-Ms technology in CC and EC therapeutic domains currently remains in early exploratory stages. Given that TAM infiltration in the TME of these two tumor types closely correlates with poor patient prognosis, and CAR-Ms technology has validated its antigen-specific phagocytosis and TME remodeling capabilities in OC and solid tumors, application of this technology in CC and EC possesses reasonable biological foundations and represents a research direction worthy of in-depth exploration in the future.

### 5.2. Microbiota and TAMs

The interplay between microbial communities and host immune systems plays a pivotal role in tumorigenesis and progression. Recent studies in various malignancies have demonstrated that the microbiota and their derived metabolites engage in a sophisticated and intimate interplay with TAMs. In colorectal cancer (CRC), *Akkermansia muciniphila* promotes M1-like TAM polarization through activation of the TLR2/NF-κB/NLRP3 signaling axis, thereby suppressing tumor growth in an NLRP3 inflammasome-dependent manner [255]. In gastric cancer (GC), depletion of butyrate-producing gut commensals reduces butyrate availability, which otherwise attenuates NF-κB and STAT3 signaling to downregulate PD-L1 and IL-10 expression in TAMs and DCs, consequently reversing the immunosuppressive microenvironment [256]. These well-characterized mechanisms in gastrointestinal malignancies establish a mechanistic precedent for understanding how microbiota modulate TAM function through metabolic and signaling pathways analogous to those governing macrophage behavior in gynecologic cancers. These regulatory patterns exhibit both shared characteristics and anatomical niche dependency across gynecological malignancies.

While the preceding sections have delineated the canonical regulatory axes, including CCL2–CCR2 and CSF1–CSF1R along with metabolic reprogramming mechanisms governing TAM function across gynecologic malignancies, emerging evidence suggests that anatomical site-specific microbiota constitute an additional layer of microenvironmental heterogeneity that modulates these established pathways. Specifically, the distinct microbial profiles of the peritoneal cavity in OC, cervicovaginal tract in CC, and endometrial cavity in EC may contribute to the divergent functional polarization of TAMs observed in these cancers, thereby extending the etiology-stratified framework beyond host-intrinsic factors to include host–microbe interactions. Based on ribonucleic acid sequencing data from The Cancer Genome Atlas OC cohort, non-human reads were extracted from tumor tissues using the Kraken2 pipeline for microbiome analysis. Acinetobacter seifertii, Achromobacter deleyi, and Microcella alkaliphila were identified as intratumoral bacterial species, with their abundances showing significant differences between immune-enriched and immune-deficient subtypes. Notably, direct evidence is currently lacking to demonstrate that Acinetobacter seifertii exhibits comparable enrichment patterns or functional effects within CC or EC, suggesting that the pro-tumorigenic role of this bacterium might be site-specific. Acinetobacter seifertii can impair antitumor immunity through inhibiting TAM migration, whereas protective flora such as Achromobacter deleyi may promote M1-like TAM activation via lipopolysaccharides [257]. In CC, Peptostreptococcus anaerobius facilitates the polarization of TAMs toward the pro-tumorigenic M2-like TAMs through the VEGF and VEGFR2 signaling axis, consequently promoting tumor angiogenesis and cell migration [258]. Atopobium vaginae is significantly enriched in EC and endometrial hyperplasia patients. This bacterium can promote tumor malignant progression by inhibiting TAM phagocytosis of tumor cells, promoting M2-like phenotype polarization, and inducing IL-6 and IL-10 secretion [259]. Research indicates that Atopobium vaginae levels are also significantly elevated in OC, and this bacterium has been verified to associate with persistent HPV infection and CC occurrence [214]. However, direct regulatory effects of Atopobium vaginae on TAMs have been established primarily in EC and OC contexts. Their functional relevance in CC remains to be further validated at the tumor tissue level. According to present evidence, microbiota dysbiosis modulates the functional phenotypes of TAMs through a multitude of mechanisms and further alters the TME in a cancer-specific manner. This regulation exhibits distinct anatomical niche dependency, with significant variations in the specific pathogenic bacteria implicated and their mechanisms of action across OC, CC, and EC. Rather than operating as independent drivers, Acinetobacter seifertii in OC, Peptostreptococcus anaerobius in CC, and Atopobium vaginae in EC appear to interface with established host pathways to shape TAM functional plasticity. These observations thus extend the etiology-stratified framework by incorporating niche-specific microbial signals.

### 5.3. Brain–Body–Tumor Tridimensional Regulation Model

**Figure 4:**
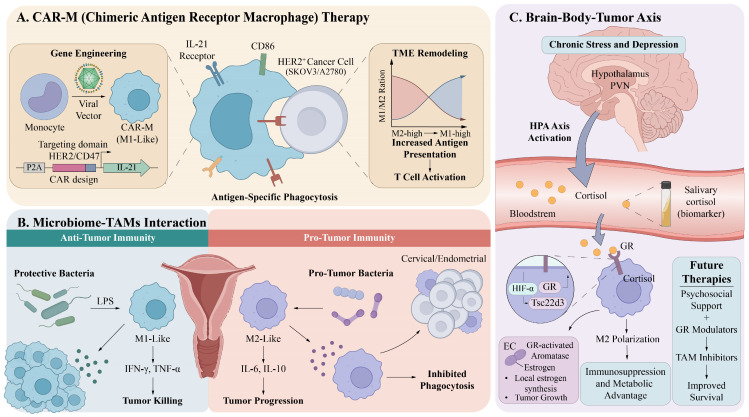
**Future therapeutic horizons targeting Tumor-Associated Macrophages (TAMs) in gynecological cancers. (A) CAR-M (Chimeric Antigen Receptor Macrophage) therapy.** Monocytes are engineered to express CARs (targeting HER2 or CD47) and co-express IL-21 to maintain a pro-inflammatory M1-like phenotype (M1-LIKE), enabling antigen-specific phagocytosis of cancer cells (SKOV3/A2780) and TME remodeling (shift towards higher M1/M2 ratio and T cell activation). **(B) Microbiome-TAMs interaction.** Protective bactria release LPS to activate M1-like TAMs, promoting IFN-γ, TNF-α, and tumor killing. Pro-tumor bacteria in the cervical and endometrial TME promote M2-like polarization and inhibit phagocytosis through IL-6 and IL-10, leading to tumor progression. **(C) Brain-Body-Tumor Axis.** Chronic stress and depression in the Brain (HPA axis PVN) activate the HPA axis, increasing systemic Glucocorticoids (Cortisol) in the Bloodstream. Salivary Cortisol is depicted as a non-invasive biomarker. In the Gynecological Cancer TME, cortisol binds to GR, upregulating HIF-1α, GR, and Tsc22d3, driving M2 Polarization and immunosuppression. Notably, in Endometrial Cancer (EC), GR activates aromatase, increasing Local Estrogen Synthesis and Tumor Growth, integrating systemic neuroendocrine monitoring with patient stratification.

Research across diverse cancer types demonstrates that the central nervous system remotely controls the TME by using neuroendocrine and metabolic signals. Extending these TAM-intrinsic developmental, signaling, and metabolic frameworks to gynecologic malignancies, recent studies specifically in OC, CC, and EC confirm that this extends our understanding of systemic tumor progression beyond local microenvironmental constraints. In this context, TAMs act as critical integrators of systemic and local signals, bringing about locally acting immunosuppression and metabolic benefits in response to neuroendocrine stress. In this regard, chronic stress and depressive emotions, as important psychosocial factors, can activate the hypothalamic–pituitary–adrenal axis (HPA axis), which is an important classical neuroendocrine pathway. When stress becomes chronic, the HPA axis becomes active, and plasma glucocorticoid levels increase through the stimulation of corticotropin-releasing hormone (CRH) neurons in the paraventricular nucleus (PVN). Notably, such neuroendocrine dysregulation constitutes general oncologic evidence across diverse malignancies rather than TAM-specific or gynecologic-specific effects. Chronic psychological stress (CPS) systematically impairs immune surveillance through sustained HPA axis and sympathetic nervous system (SNS) overactivation, wherein elevated glucocorticoids and catecholamines compromise CD8^+^ T cell and NK cell cytotoxicity while promoting immunosuppressive MDSCs and TAMs, establishing a self-perpetuating oncogenic ecosystem via multisystem crosstalk [260]. The process results in the significant upregulation of mRNA expression levels of HIF-1α, glucocorticoid receptor (GR), and Tsc22d3 in TAMs that drive the polarization of TAM to the M2-like phenotype, which worsens tumor progression [261]. This GR-mediated polarization converges with the hypoxia-driven metabolic pathways previously characterized, suggesting that systemic stress amplifies local immunosuppressive mechanisms through shared HIF-1α signaling. A cohort study involving 85,327 individuals with gynecological cancers indicated that mortality and risk of death were significantly higher in individuals with incident depression following the diagnosis of OC, CC, and EC. The mechanisms include dysregulation of the HPA axis and reduced treatment adherence [1]. Epidemiological studies suggest that the prevalence of depression and anxiety in OC patients is significantly higher than those in the healthy female population, and chronic stress can aggravate disease through the neuroendocrine pathway [262]. Psychological stress states such as anxiety and depression can elevate salivary cortisol levels in EC patients, subsequently activating aromatase in the TME through GR-mediated mechanisms, promoting local estrogen synthesis and accelerating malignant tumor progression [263]. This mechanism directly links systemic neuroendocrine signals to the hormone-dependent TAM regulation and ERRα metabolic axis previously described in endometrial cancer, further illustrating how systemic factors contextualize the cancer-specific TAM biology outlined above. Before clinical translation, future research must elucidate how chronic glucocorticoid exposure intersects with the established CCL2–CCR2 and CSF1–CSF1R signaling axes to influence therapeutic resistance. Prospective studies should validate whether salivary cortisol serves as a surrogate marker for TAM functional status in the context of the precision medicine frameworks discussed above, thereby determining whether systemic neuroendocrine monitoring can complement existing biomarker strategies for patient stratification (Figure 4).

## 6. Discussion and Conclusions

This review synthesizes current evidence on the dual regulatory patterns of TAMs in OC, CC, and EC through a cross-cancer comparative framework. This framework reveals a fundamental tension in gynecologic TAM biology in which shared molecular machinery generates divergent functional outputs due to distinct etiological constraints. While the CCL2–CCR2 and CSF1–CSF1R signaling axes and metabolic reprogramming mechanisms are conserved across all three malignancies, anatomical location specificity in OC, pathogen-driven mechanisms in CC, and hormone-dependent characteristics in EC give TAMs unique functional polarization features. This dialectical relationship between common foundations and tumor-specific characteristics offers insights into shared mechanisms that may underlie the limited efficacy of current immune checkpoint inhibitors in gynecological malignancies while also providing cellular and molecular bases for understanding different therapeutic responses across distinct cancer types. For translational application, while macrophage-targeting strategies may be broadly applicable across these cancers, patient stratification must account for cancer-specific TAM ontogeny, particularly the ontogenically restricted CD163^+^Tim4^+^ subset in OC, which offers limited cross-cancer translational value. Network-dependent interactions formed among TAMs, mesothelial cells, cancer-associated fibroblasts, and T cells, rather than purely cell-autonomous defects, may be the primary reason for limited clinical responses to existing targeted strategies.

The network-dependent interactions between TAMs and other cells within the TME constrain the clinical efficacy of current targeted therapeutic strategies. Therefore, future investigations should prioritize network-based combination therapies. At the translational medicine level, intervention strategies targeting TAMs have evolved from simple cell depletion to multi-dimensional precise regulation. Strategies blocking TAM recruitment (including CSF1R or CCR2 inhibitors), metabolic reprogramming (including targeting PI3K or lactate metabolism), and phenotypic reversal (including nanoparticle delivery of IRF5 mRNA) have demonstrated potential for overcoming immune therapeutic resistance in preclinical models. However, challenges remain. The significant heterogeneity of TAMs in terms of their developmental origins, spatial localization, and polarization states limits the effectiveness of targeting one pattern. In addition, despite the ability to enhance antitumoral immunity in association with PD-1 inhibitors, the urgent need for biomarker-based patient stratification systems is driven by the frequent occurrence of high-grade adverse events. Prognostic and therapy-guiding capacities of the density of CD163-positive M2-like TAMs, proportions of circulating intermediate monocytes, tertiary lymphoid structure (TLS)-related characteristics, and cross-cancer standardized assessment systems have yet to be established.

Research remains methodologically and mechanistically limited. Many studies use bulk analysis of single-timepoint samples without spatiotemporal dynamics in single-cell resolution. Evidence suggesting that TME metabolic heterogeneity dictates macrophage phenotypic plasticity is scarce. The interaction mechanisms between TAMs and microbiota (for example, Atopobium vaginae or Peptostreptococcus anaerobius) or neuroendocrine signals (the brain–body–tumor axis) are still being explored. Future research should combine spatial transcriptomics, metabolomics, and artificial-intelligence-assisted analysis to construct spatiotemporal evolution atlases of TAM functional states. Addressing these networks should also focus on the ternary interaction networks formed by TAMs and stromal cells with an emphasis on TAMs, as well as cancer-associated fibroblasts and T cells and the development of combined intervention protocols designed to target nodes in the networks. The emergence of chimeric antigen receptor macrophage (CAR-M) technology, probiotic intervention, and stress management techniques offers innovative approaches to reprogramming immunosuppressive TMEs beyond drug development.

TAMs are the key regulatory nodes in the TME of gynecological malignancies. Their developmental plasticity, metabolic plasticity, and intercellular communication network form the basis of tumor progression and therapeutic resistance. Importantly, ontogenic and anatomical heterogeneity result in cancer-specific compositions of TAM subpopulations, with certain subsets being restricted to particular microenvironments and absent in others, suggesting the need for etiology-stratified therapeutic approaches. A comprehensive analysis of common cross-cancer mechanisms and microenvironment-dependent specificities in TAMs, coupled with the establishment of individualized TAM-targeted therapeutic systems based on molecular classification, may offer valuable insights for addressing current immunotherapeutic limitations in gynecology. To improve the prognosis of patients with malignant tumors, future clinical translation must balance efficacy and safety, guiding patient stratification through multi-omics biomarkers to advance TAM research toward clinical precision.

## Data Availability

No new data were created or analyzed in this study. Data sharing is not applicable.
